# SEMA6B Overexpression Predicts Poor Prognosis and Correlates With the Tumor Immunosuppressive Microenvironment in Colorectal Cancer

**DOI:** 10.3389/fmolb.2021.687319

**Published:** 2021-12-06

**Authors:** Tiegang Li, Zheng Yan, Weiqi Wang, Rixin Zhang, Wenqiang Gan, Silin Lv, Zifan Zeng, Yufang Hou, Min Yang

**Affiliations:** State Key Laboratory of Bioactive Substances and Function of Natural Medicine, Institute of Materia Medica, Chinese Academy of Medical Sciences and Peking Union Medical College, Beijing, China

**Keywords:** SEMA6B, colorectal cancer, prognosis, tumor microenvironment, immune response, immune checkpoint

## Abstract

**Background:** Semaphorin 6b (SEMA6B) is a member of the semaphorin axon-guidance family and has been demonstrated to both induce and inhibit tumor progression. However, the role of SEMA6B in colorectal cancer (CRC) has remained unclear. This study sought to explore the promising prognostic biomarker for CRC and to understand the expression pattern, clinical significance, immune effects, and biological functions of SEMA6B.

**Methods:** SEMA6B expression in CRC was evaluated *via* multiple gene and protein expression databases and we identified its prognostic value through The Cancer Genome Atlas (TCGA) and Gene Expression Omnibus (GEO) databases. Correlations between SEMA6B expression and components of the tumor immune microenvironment were analyzed by packages implemented in R, Tumor Immune Estimation Resource (TIMER), Gene Expression Profiling Interactive Analysis (GEPIA), and Tumor-Immune System Interactions database (TISIDB). RNA interference was performed to silence the expression of SEMA6B to explore its biological roles in the colon cancer cell lines HCT116 and LoVo.

**Results:** The messenger RNA (mRNA) level of SEMA6B and the protein expression were higher in CRC tissues than adjacent normal tissues from multiple CRC datasets. High SEMA6B expression was significantly associated with dismal survival. Multivariate Cox regression analysis demonstrated that SEMA6B was an independent prognostic factor for progression-free survival (PFS). The nomogram showed a favorable predictive ability in PFS. Functional enrichment analysis and the Estimation of STromal and Immune cells in MAlignant Tumor tissues using Expression data (ESTIMATE) algorithm revealed that the gene cluster associated with the high SEMA6B group were prominently involved in immune responses and inflammatory activities. Notably, SEMA6B expression was positively correlated with infiltrating levels of CD4^+^ T cells, macrophages, myeloid-derived suppressor cells (MDSCs), regulatory T cells (Tregs), neutrophils, and dendritic cells. Moreover, SEMA6B expression displayed strong correlations with diverse marker sets of immunosuppressive cells in CRC. Integrative analysis revealed that immunosuppressive molecules and immune checkpoints were markedly upregulated in CRC samples with high SEMA6B expression. Furthermore, knockdown of SMEA6B in colon cancer cells significantly inhibited cell proliferation, migration, invasion and reduced the mRNA levels of immunosuppressive molecules.

**Conclusion:** Our findings provide evidence that high SEMA6B expression correlated with adverse prognosis and the tumor immunosuppressive microenvironment in CRC patients. Therefore, SEMA6B may serve as a novel prognostic biomarker for CRC, which offers further insights into developing CRC-targeted immunotherapies.

## Introduction

Colorectal cancer (CRC) is a major cause of cancer mortality around the word. Approximately 20% of patients occurred metastases at diagnosis (Garborg et al., 2013; Siegel et al., 2014). Although many advances in systemic therapy and liver-directed treatments made thus far, 5 years survival rate is only 12–14% in patients with metastatic CRC (Siegel et al., 2017; Bray et al., 2018). Over the past few years, full recognition of the complex interactions between cancer cells and the immune system has led to a rapid development in immunotherapeutic approaches. Immunotherapeutic strategies include immune checkpoint inhibitors, cancer vaccines, adoptive cell transfer, oncolytic viral therapy, and carcinoembryonic antigen (CEA) T-cell bispecific antibodies, which focus on selectively enhancing the host immune system to fight cancer (Kakimi et al., 2017; Szeto and Finley 2019; Wrobel and Ahmed 2019).

Immune checkpoint inhibitors currently represent the main domain of immunotherapy and have achieved clinical benefits to patients with advanced cancer including renal cell carcinoma, non-small cell lung cancer and malignant melanoma (Reck et al., 2016; Atkins et al., 2017; Gettinger et al., 2018; Ribas and Wolchok 2018). Recent success in using antibodies against various immune checkpoints such as cytotoxic T-lymphocyte-associated antigen 4 (CTLA-4), programmed death 1 (PD-1), and programmed death ligand 1 (PD-L1) for cancer immunotherapy has brought this approach being implemented as a new treatment modality for CRC, especially in terms of targeting the microsatellite instability-high (MSI-H) phenotype (Le et al., 2015; Overman and McDermott 2017; Overman et al., 2018; Le et al., 2020). However, clinical immunotherapeutic trials have revealed that anti-CTLA-4 monoclonal antibodies (mAbs) yield unsatisfactory clinical efficacies in unselective CRC patients (Chung et al., 2010), and anti-PD-L1 mAbs and anti-PD-1 have shown little or no response rates in metastatic CRC (mCRC) (Le, Uram and Wang 2015; Overman and McDermott 2017). Although there is clear clinical evidence for a therapeutic role of immune checkpoint inhibitors in deficient mismatch repair (dMMR) or MSI-H mCRC, the majority of mCRC patients with proficient MMR (pMMR) or microsatellite stable (MSS) phenotypes do not benefit from this type of immunotherapy (Koi and Carethers 2017; Ciardiello and Vitiello 2019; Ganesh et al., 2019; Judge et al., 2020; Morse et al., 2020). Furthermore, previously described molecular features, such as immunoscore, PD-1, PD-L1, MSI, mutational load, and consensus molecular subtypes have not been identified in predicting responses to immune checkpoint inhibitors based on immunotherapy. (Emambux and Tachon 2018; Sveen et al., 2020). Therefore, it is required to discover novel biomarkers with latent prognostic value and screen immune-based therapeutic targets for CRC patients.

Semaphorin family members were initially characterized as axon-guidance factors with functions in axonal navigation, but have subsequently also been linked to the pathology of various diseases, such as cancer, immune disease and neurodegenerative disease (Műzes and Sipos 2014; Neufeld and Mumblat 2016; Franzolin and Tamagnone 2019). Accumulated studies have shown that some semaphorins—including semaphorin 3E (SEMA3E), SEMA4D, SEMA5A, SEMA6D, and SEMA7A—play vital roles in tumorigenesis and tumor development by promoting angiogenesis and tumor-cell migration, as well as the epithelial-mesenchymal transition (EMT); in contrast, SEMA3A, SEMA3B, and SEMA3F exhibit tumor-inhibitory effects (Neufeld et al., 2012; Neufeld and Mumblat 2016; Gurrapu and Tamagnone 2019). Mechanisms that account for the diversity of semaphorin signaling responses in different cellular contexts can profoundly affect these different biological activities. SEMA6B, a member of the semaphorin axon-guidance family, has recently been investigated in terms of human SEMA6B gene expression and its roles in cancer. In breast cancer tissues, the SEMA6B promoter undergoes abnormal methylation, and downregulation of SEMA6B messenger RNA (mRNA) has been found in tumor samples (D'Apice and Costa 2013; Kuznetsova et al., 2007). In CRC patients, miR-30b could mediate axon guidance and is significantly negatively correlated with SEMA6B ( Coebergh van den Braak et al., 2018 ). Among different human cell lines, high levels of SEMA6B mRNA have been observed in MCF-7 breast adenocarcinoma cells, and these levels have been found to be downregulated by 9-cis-retinoic acid, an anti-proliferative and differentiation-promoting agent (Murad and Collet 2006). Functionally, SEMA6B has been found to exert complex roles in the development and progression of tumors such as breast cancer (D'Apice and Costa 2013; Murad and Collet 2006), glioblastoma (Kigel and Rabinowicz 2011), gastric cancer (Ge and Li 2013), and testicular cancer (Ji and Wang 2020). However, the prognostic value of SEMA6B in CRC and the relationship between SEMA6B and immune responses remain elusive.

At present study, we assessed SEMA6B expression and clarified its potential prognostic value in CRC patients using The Cancer Genome Atlas (TCGA), Human Protein Atlas (HPA), and Gene Expression Omnibus (GEO) databases. Moreover, we explored the underlying biological functions and relevant pathways of SEMA6B and investigated correlations of SEMA6B with a variety of tumor-infiltrating immune cells (TIICS) as well as tumor-immunity status *via* comprehensive bioinformatic analyses. Taken together, our present findings may help to uncover prominent immunoregulatory roles of SEMA6B in the CRC microenvironment, and provide a promising biomarker and target for CRC diagnosis and immunotherapy.

## Materials and Methods

### Data Resources

RNA-sequencing data for the TCGA-colon adenocarcinoma (TCGA-COAD) and TCGA-rectal adenocarcinoma (TCGA-READ) cohorts, including 638 CRC samples and 51 normal tissue samples, were downloaded from public databases (https://portal.gdc.cancer.gov/). Corresponding clinicopathological characteristics for each patient—including age, gender, race, tumor location, disease type, tumor stage, tumor-node-metastasis (TNM) classification, venous invasion, lymphatic invasion, pretreatment CEA level, and survival information—were also retrieved from the TCGA data portal. Only patients with both survival information and expression data were included in the present study. Another mRNA expression profile for 308 normal tissue samples was obtained in transcripts-per-million (TPM) format from the Genotype-Tissue Expression (GTEx) project (https://www.gtexportal.org/home/datasets), which is another large-scale repository cataloging gene expression from healthy individuals. Then, Ensembl gene IDs were mapped to human gene SYMBOL in terms of GENCODE V22 annotations for human datasets through the use of R/Bioconductor packages. As the raw mRNA sequence datasets from TCGA were normalized in terms of fragments per kilobase million (FPKM) *via* log_2_(FPKM+1), these datasets were scaled to a total depth of 10^6^ fragments per sample and were interpreted as TPM in order to more easily compare the proportion of reads that was aligned to a given gene in each sample. Subsequently, any gene with a mean expression of ≤0.3 across all samples was deleted from the final mRNA expression matrices for subsequent analysis. Six independent datasets from the GEO database were used for external validation in the present study, including GSE41258, GSE44076, GSE37182, GSE20842, GSE83889, and GSE39582, together with survival information. A normalized expression matrix from GEO database was applied directly for the analyses. The protein expression levels of SEMA6B in clinical specimens from CRC patients and normal tissues were examined using immunohistochemical data from the HPA database (http://www.proteinatlas.org/). Since the data used in the present study were provided by TCGA and GEO, informed consent or ethical approval was not required. Furthermore, the present study fully adhered to all TCGA publication guidelines.

### Survival Analysis

Kaplan-Meier (KM) curves were plotted to compare overall survival (OS), disease-free survival (DFS), progression-free survival (PFS), and relapse-free survival (RFS). These curves were generated with an optimum cut-off value for SEMA6B mRNA expression using the survfit function from the R package ‘survminer’, and a log-rank test was conducted to compare differences between survival status. Univariate and multivariate analyses of Cox proportional-hazards regression models were performed to obtain hazard ratios (HRs) with 95% confidence intervals (CIs) and statistical significance; the results were illustrated using a forest plot *via* GraphPad Prism 8.0. PFS-related nomogram models were established based on the multivariate Cox regression results. Calibration curves were drawn, and the concordance index (C-index) was computed to assess the prediction power of the nomogram.

Additionally, the prognostic values of SEMA6B expression in breast, esophageal, stomach, liver, lung, and ovarian cancers were assessed by the best cut-off values *via* Kaplan-Meier plotter (www.kmplot.com). HRs with 95% CIs and log-rank *p* values were also computed on the Kaplan-Meier plotter web page.

### DNA Mutation and Methylation Analyses

To investigate the regulation of expression associated with the expression profile of SEMA6B, DNA mutation and methylation analyses were explored *via* online databases. Specifically, somatic mutation information was identified by the cBioPortal platform (www.cbioportal.org), which is a comprehensive web resource for exploring, visualizing, and analyzing multidimensional cancer-genomic data. Methylation changes in SEMA6B in CRC and adjacent normal tissues were compared using UALCAN (http://ualcan.path.uab.edu/index.html) (Chandrashekar et al., 2017) and Wanderer (http://maplab.imppc.org/wanderer/) (Díez-Villanueva et al., 2015) databases, which are web tools that can be employed to analyze DNA methylation profiles and gene expression from TCGA.

### ROC Analysis

Receiver operating characteristic (ROC) analysis was used to evaluate the diagnostic accuracy for both OS and PFS; areas under the curve (AUCs) as well as *p* values were calculated *via* SPSS 25.0 software.

### Identification of Differentially Expressed Genes

In accordance with the optimum cutoff value in KM survival analysis for OS, patients were classified into two groups (low and high SEMA6B expression) across TCGA datasets. Linear models were used to screen differentially expressed genes (DEGs) between these two groups by using the R package, “limma”. The threshold for identifying DEGs was set as the false discovery rate (FDR)-adjusted *p* value <0.01 and absolute value of log2 (fold change) ≥ 1. With the R package, “gghplot2”, a volcano plot was generated to visualize fold changes and *t*-test criteria.

### GO and KEGG Pathway Enrichment Analyses of DEGs

Gene Ontology (GO) and Kyoto Encyclopedia of Genes and Genomes (KEGG) enrichment analyses using 1,789 overexpressed DEGs were performed using R software with the aid of the “clusterProfiler” package. Biological processes (BP) and molecular functions (MF) were included in the GO enrichment analysis. Only terms with an FDR-adjusted *p* value <0.01 were considered to be statistically enriched. The top-15 enriched terms ordered by q value, from small to large, are shown in the corresponding plot.

### Gene Set Enrichment Analysis of DEGs

GSEA (version 4.1.0) was used to evaluate correlations between SEMA6B expression (high vs low) using the TCGA dataset. The annotated gene set was c2. cp.kegg.v6.2. symbols.gmt. Standard settings with 1,000 runs of gene permutations were employed for each analysis to determine the enriched pathways. Normalized enrichment scores (NES) and FDR-adjusted *p* values were obtained to indicate significantly enriched gene sets and pathways.

### Gene Set Variation Analysis and Functional Annotation

To investigate the difference on biological pathways and processes according to the expression patterns of SEMA6B, GSVA was employed with the “GSVA” R package. GSVA is a non-parametric unsupervised method to explore the variation of pathway activity over samples (Hänzelmann et al., 2013). The annotated gene set was also “c2. cp.kegg.v6.2. symbols” downloaded from the MSigDB database (https://www.gsea-msigdb.org/gsea/index.jsp). An adjusted *p* value ≤0.05 was considered statistically significance. A heatmap was drawn to display the enriched score value of each sample using the “pheatmap” R package.

### Generation of Immune and Stromal Scores

The Estimation of STromal and Immune cells in MAlignant Tumor tissues using Expression data (ESTIMATE) algorithm (Yoshihara and Shahmoradgoli 2013) was applied to calculate immune scores, stromal scores, ESTIMATE scores, and tumor purity for each patient from the TCGA dataset *via* R software loaded with the “estimate” package with default parameters.

### TIMER Database Analysis

The Tumor Immune Estimation Resource (TIMER) is a web-based platform for systematic analysis of immune infiltrates across diverse cancer types from the TCGA (http://timer.comp-genomics.org/) (Li et al., 2017), which adopts a deconvolution of previously published computational approaches to infer TIICs from gene expression profiles. In the present study, correlations between SEMA6B expression and TIICs (B cells, CD4^+^T cells, CD8^+^T cells, neutrophils, macrophages, and dendritic cells) were investigated separately in TCGA-COAD and TCGA-READ. Meanwhile, correlations between SEMA6B expression and gene markers of TIICs were also analyzed *via* the “Gene_Corr” module.

### Tumor-Immune System Interactions Database Analysis

To further determine the relationship between SEMA6B mRNA expression and the abundance of TIICs, the Tumor-Immune System Interactions database (TISIDB) was used to determine the correlation between SEMA6B mRNA expression and tumor-infiltrating lymphocytes (TILs). TISIDB is a web portal for assessing tumor and immune system interactions, which integrates multiple heterogeneous data types (http://cis.hku.hk/TISIDB/index.php) (Ru et al., 2019).

### Gene Correlation Analysis

The Gene Expression Profiling Interactive Analysis (GEPIA2) online database (http://gepia2.cancer-pku.cn/#index) (Tang et al., 2019) was used to determine correlations between SEMA6B mRNA expression and gene markers of TIICs in CRC and adjacent normal tissues. Pearson correlation coefficients between SEMA6B expression and immunoinhibitor gene sets were visualized using R software with the “corrplot” package.

### Heatmaps and Hierarchical Clustering Analysis

The “complexHeatmap” package from Bioconductor was used to plot heatmaps in terms of the expression of immunosuppressive gene sets as well as immune and stromal scores in different subgroup samples with R software. Specifically, Z-score normalization was applied in the expression dataset matrix, and then Euclidean distance was used to determine hierarchically clustered.

### Analysis of Cell Viability

The mRNA expression level of SEMA6B in different colon cell lines was examined using the Cancer Cell Line Encyclopedia (CCLE) website ( https://sites.broadinstitute.org/ccle ). According to the survey, we selected two cell lines with high SEMA6B levels for subsequent research, i.e., HCT116 and LoVo cells.

A cell-counting kit 8 (CCK8) was used to determine cell proliferation. HCT116 and LoVo cells were plated and cultured in a 96-well plate with 1,500 cells per well, and then were interfered with by SEMA6B- and NC-siRNA for 0, 24, 48, or 72 h. After the interference, the supernatant was removed, and 100 μl DMEM or DMEM F12K was added in the presence of 10 μl of CCK8, and the cells were incubated at 37°C for 4 h. Then, the absorbance at 450 nm was assayed.

### Cell Migration and Invasion Assay

Cells were transfected with NC- or SEMA6B-siRNA for 48 h. An invasion chamber with 8 μ pores (Matrigel invasion chamber; Corning, Corning, NY, United States) was used to evaluate the potency of cells in the migration and invasion stages. For the invasion assay, 2 × 10^5^ cells in serum-free medium were added to the upper chamber. Then, 500 μl of 10% FBS DMEM or DMEM F12K was added to the lower chamber, and the number of cells that had migrated after 48 h was quantified by counting five random fields under a microscope (IX70; Olympus Corp., Tokyo, Japan). Similar methods were adopted for the migration assay, except that 1 × 10^5^ cells were added to the upper chamber without the Matrigel coating. Five random fields were counted for each chamber.

### RNA Extraction and Real-Time Polymerase Chain Reaction

Total RNA was isolated from cells using an RNA isolation kit (Beyotime, Shanghai, China) following the manufacturer’s instructions. The concentration of total RNA was quantified using a microplate reader; then, 1 μg of total RNA was reverse-transcribed to complementary DNA using the PrimeScript RT reagent kit (Takara Bio, Kusatsu, Japan). Quantitative real-time PCR (qRT-PCR) was applied using a TB Green Premix Ex Taq II kit (Takara Bio). Glyceraldehyde-3-phosphate dehydrogenase (GAPDH) was used as an internal control. Each experiment was performed in triplicate. Differences in gene expression level, expressed as fold changes, were calculated using the 2^−ΔΔCt^ method. The following primers were used: interleukin 6 signal transducer (IL6ST), forward: 5′-GGA​AGC​TCA​GCC​AAC​TCG​AA-3′, reverse: 5′-CCC​AAG​CAG​CCT​TTC​CAT​GA-3′; B and T lymphocyte attenuator (BTLA), forward: 5′-CTG​AGG​TTT​TGT​GGT​GGA​GAG​A-3′, reverse: 5′-TTG​CAC​CCC​CAA​ATC​TAA​GGA-3′; CD274 (PD-L1), forward: 5′-GGA​AAT​TCC​GGC​AGT​GTA​CC-3′, reverse: 5′-TGA​CAG​CTG​GTG​GCA​TTC​AA-3′; Galectin 1 (LGALS1), forward: 5′-CGC​TAA​GAG​CTT​CGT​GCT​GA-3′, reverse: 5′- CGT​TGA​AGC​GAG​GGT​TGA​AG-3′; interleukin 1, beta (IL1B), forward: 5′-CCT​GAG​CTC​GCC​AGT​GAA​AT-3′, reverse: 5′-GTC​GGA​GAT​TCG​TAG​CTG​GA-3′; intercellular adhesion molecule 1(ICAM1), forward: 5′-GGC​CCC​ACA​GAC​TTA​CAG​AA-3′, reverse: 5′-RGTCAGGAAGTGTGGGCCTTT-3′; hepatitis A virus cellular receptor 2 (HAVCR2), forward: 5′- CTA​CTG​CTG​CCG​GAT​CCA​AA-3′, reverse: 5′- GTC​CCC​TGG​TGG​TAA​GCA​TC-3′; endothelin receptor type B (EDNRB), forward: 5′- AGG​TGC​TAT​CGT​TCA​ACT​TCA-3′, reverse: 5′- TAG​CCA​CTT​TAG​GCA​ACC​AA-3′; and transforming growth factor β2 (TGF-β2), forward: 5′-TAC​CAC​CTT​TCC​GAT​TGC​CC-3′, reverse: 5′-TGG​CCT​GAC​TCT​TGT​GCT​TT-3′.

### Statistical Analysis

Data analysis and visualization were performed using R software (version 4.0.0) with appropriate packages, as well as with SPSS25.0 (IBM Corp., Armonk, New York, United States) and GraphPad Prism 8.0 (GraphPad Software Inc., San Diego, CA, United States). Pearson’s correlations were used to analyze pairwise relationships between mRNA levels of different genes. For continuous variables, differences among groups were analyzed using one-way analysis of variance (ANOVA) for comparisons of more than two groups, and *via* t-tests for comparisons between only two groups. Chi-squared tests (χ ^2^) were used to evaluate associations between SEMA6B expression and clinicopathological parameters. A two-sided *p* < 0.05 was considered statistically significant.

## Results

### SEMA6B Expression is Upregulated in CRC Tissues

**FIGURE 1:**
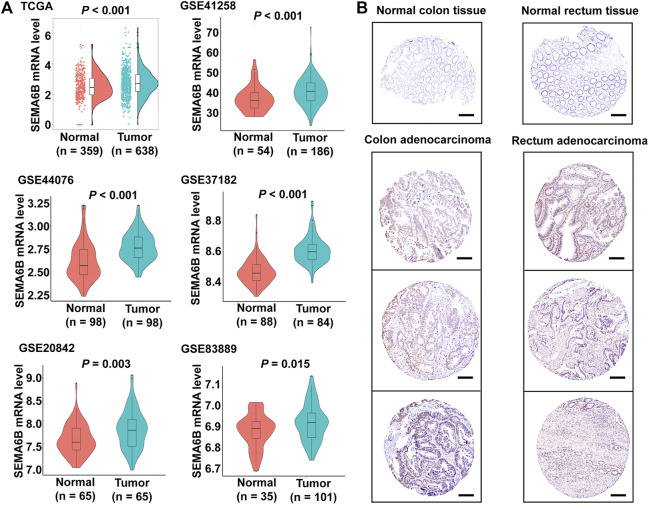
SEMA6B mRNA and protein expression in CRC tissues and normal tissues. **(A)** The expression of SEMA6B mRNA in normal and tumor samples derived from TCGA and GEO databases. **(B)** Representative immunohistochemical images of SEMA6B in CRC tissues and corresponding normal tissues using the HPA database. Scale bars = 200 µm.

Using RNA-sequencing data from the TCGA-CRC dataset and GTEx project, we found that SEMA6B mRNA expression in CRC tissues (n = 638) was significantly higher than that in normal colorectal tissues (n = 359; *p* < 0.001). Meanwhile, upregulated SEMA6B mRNA levels in CRC tissues were also validated in GSE datasets, including GSE41258 (*p* < 0.001), GSE44076 (*p* < 0.001), GSE37182 (*p* < 0.001), GSE20842 (*p* = 0.003), and GSE83889 (*p* = 0.015). (Figure 1A). Moreover, immunohistochemical staining obtained from the HPA database demonstrated that protein expression levels of SEMA6B were consistent with their transcriptional levels in comparison with those in normal tissues (Figure 1B). We further detected the function of methylation in regulating the expression of SEMA6B and found that the DNA methylation levels of SEMA6B were dramatically downregulated in CRC tissues compared with those in normal colorectal samples (*p* < 0.001) using the UALCAN web tool (Supplementary Figure S1A). As shown in Supplementary Figure S1B, data from the Wanderer web tool were similar (*p* < 0.05; normal n = 44, tumor n = 400), with most of the SEMA6B probes in the 450 methylation array exhibiting significant differences between CRC and normal specimens. Otherwise, few mutational or somatic copy-number alterations of SEMA6B were observed in CRC tissues (Supplementary Figure S2).

### High SEMA6B Expression Predicts Poor Prognosis of CRC Patients

**FIGURE 2:**
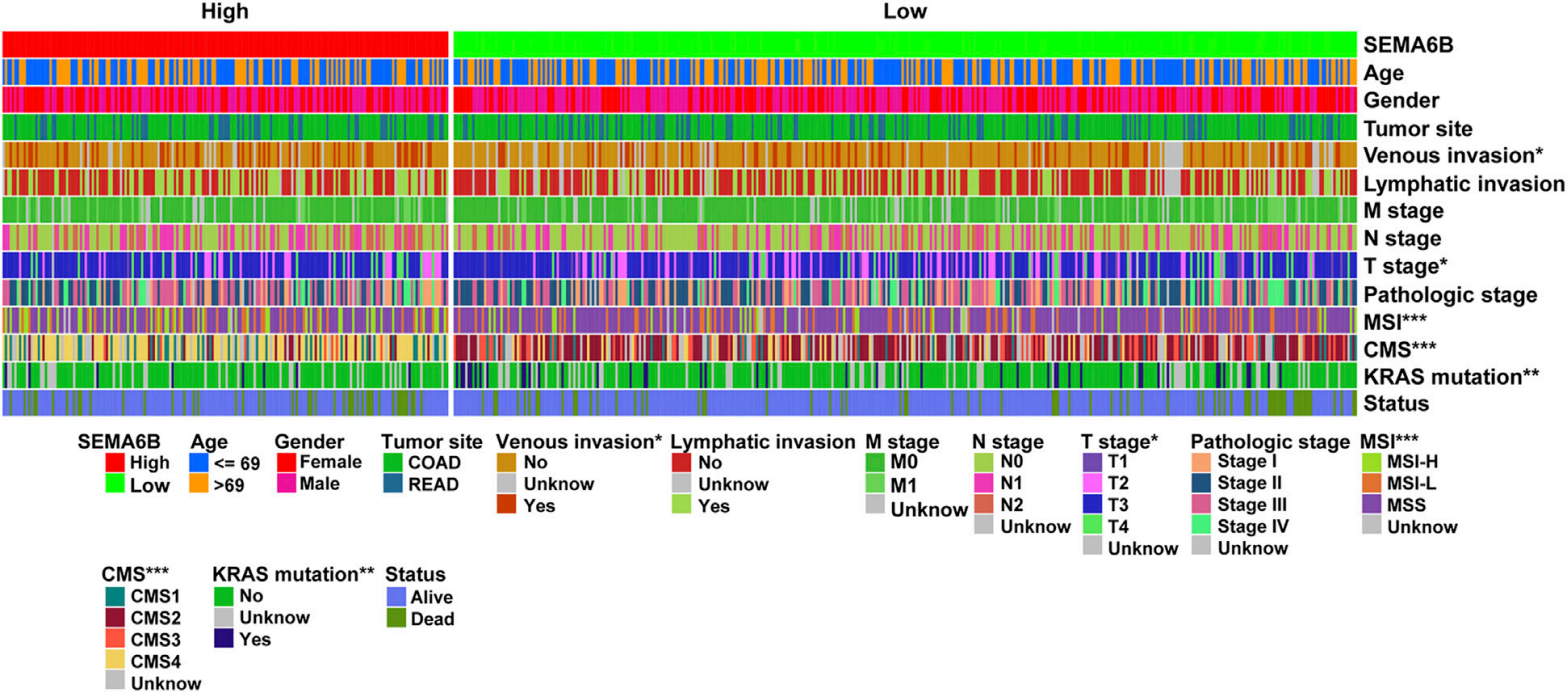
Cluster heatmap of correlations between SEMA6B and molecular subtypes in the TCGA dataset. The relationships between SEMA6B level and each clinicopathological characteristic were measured with the χ^2^ test. *p < 0.05, **p < 0.01, and ***p < 0.001.

We next assessed associations of SEMA6B expression with clinicopathological features of CRC patients using the TCGA-CRC dataset. As shown in Figure 2, the expression levels of SEMA6B were significantly associated with venous invasion, T stage, MSI, KRAS mutation, and CMS. However, SEMA6B expression in CRC was not significantly correlated with other clinicopathological characteristics, including age, gender, tumor site, lymphatic invasion, M stage, N stage, pathological stage, and patient status.

**FIGURE 3:**
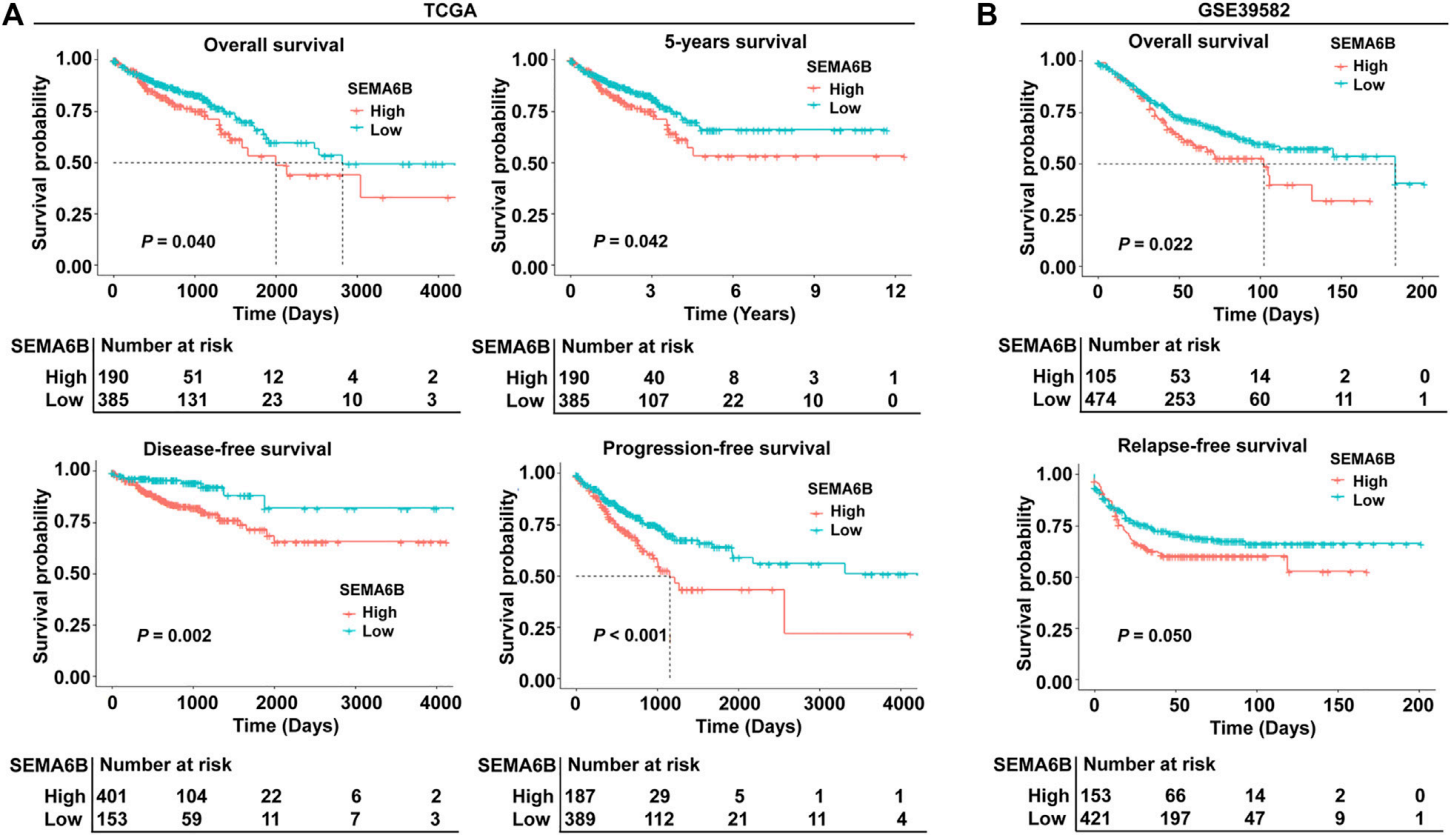
Kaplan-Meier survival analysis comparing high and low expression of SEMA6B in patients with CRC. **(A)** Survival curves of overall survival (OS), 5 year survival, disease-free survival (DFS), and progression-free survival (PFS) in the TCGA dataset. **(B)** Survival curves of OS and relapse-free survival (RFS) in the validated GEO dataset (GSE39582).

To evaluate the prognostic value of SEMA6B, CRC patients were divided into high and low SEMA6B expression groups according to the optimal cut-off value of SEMA6B levels. Kaplan-Meier survival curve analysis showed that CRC patients with high SEMA6B expression had shorter OS (*p* = 0.040), 5 year survival (*p* = 0.042), DFS (*p* = 0.002), and PFS (*p* < 0.001) compared to those with low SEMA6B expression (Figure 3A). To validate these findings, we analyzed the prognostic significance of SEMA6B using another GEO cohort (GSE39582). In line with results in the TCGA dataset, the high SEMA6B expression group exhibited poor survival (OS: *p* = 0.022; RFS: *p* = 0.050) (Figure 3B). Subsequently, the Kaplan-Meier plotter database was used to analyze the prognostic potential of SEMA6B in different cancers. Interestingly, high SEMA6B expression levels were associated with poor prognosis of OS in lung adenocarcinoma and lung squamous cell carcinoma (Supplementary Figure S3), while SEMA6B expression had less influence on the prognoses within other types of cancers (Supplementary Figure S3).

**FIGURE 4:**
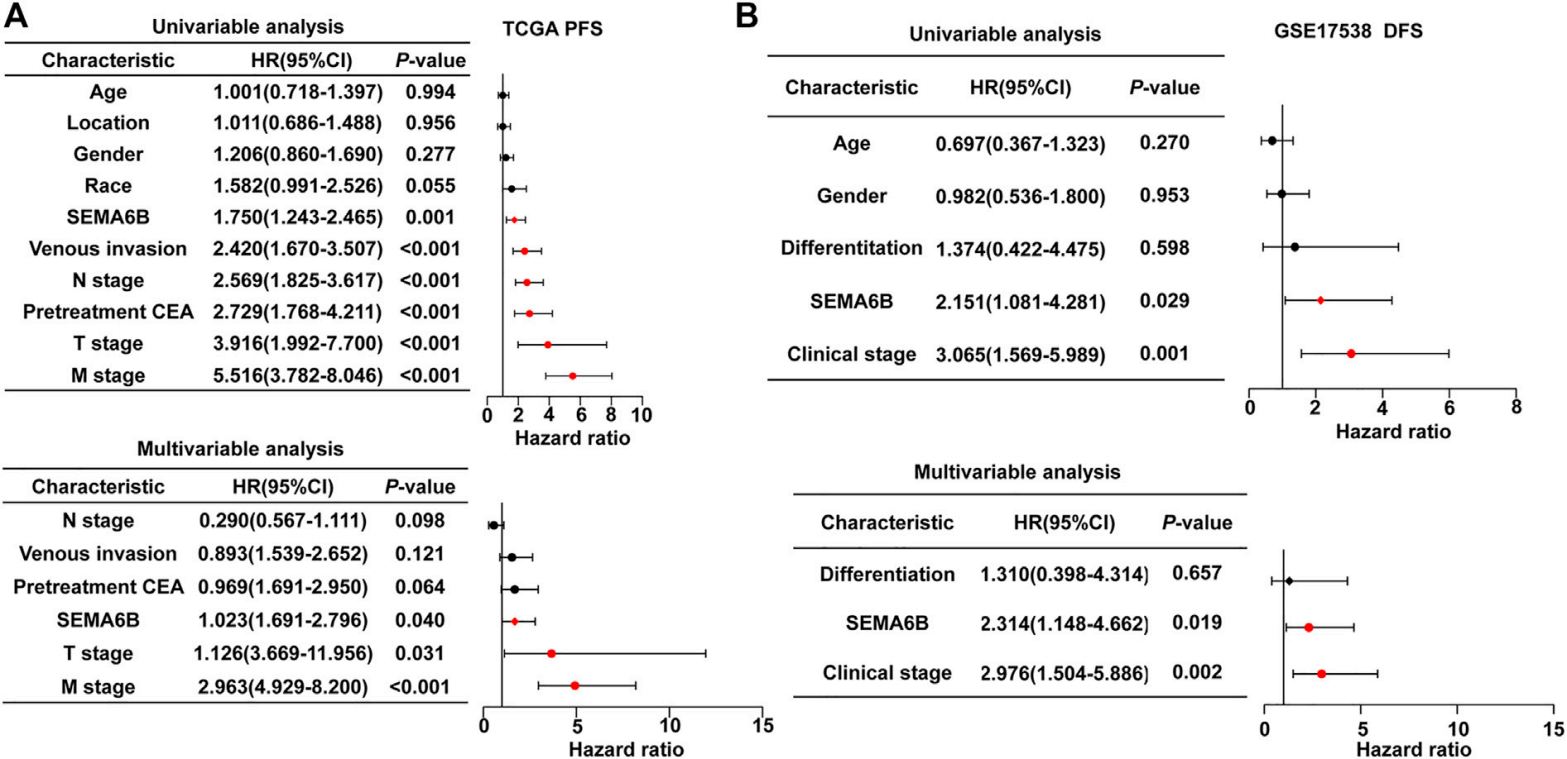
Univariate and multivariate Cox regression analyses to evaluate the prognostic value of the SEAM6B in CRC patients. **(A)** PFS for TGGA dataset. **(B)** DFS for GSE17538 dataset. Forest plots visualizing the hazard ratios (HRs) and 95% confidence intervals (CI) for each variable. Differences with *p* < 0.05 were considered significant.

Furthermore, to explore the clinical prognostic significance of SEMA6B in CRC, Cox regression analysis was performed to determine PFS and OS. Univariate Cox regression analysis showed that SEMA6B, venous invasion, N stage, pretreatment CEA, T stage, and M stage were significantly associated with PFS in CRC patients sourced from the TCGA dataset (*p* < 0.05; Figure 4A). Meanwhile, multivariate Cox regression analysis showed that SEMA6B, T stage, and M stage were independent prognostic factors for PFS among CRC patients (*p* < 0.05; Figure 4A). For OS, univariate Cox regression analysis showed that SEMA6B, age, venous invasion, pretreatment CEA, T stage, N stage, and M stage have statistical significance; however, SEMA6B expression was not an independent prognostic factor in the multivariate analysis for OS (*p* > 0.05; Supplementary Figure S4). Moreover, in the GSE17538 validation CRC dataset, SEMA6B was an independent prognostic factor for DFS in multivariate Cox regression analysis (*p* < 0.05; Figure 4B).

### Prognostic Nomogram Models Based on SEMA6B for CRC

**FIGURE 5:**
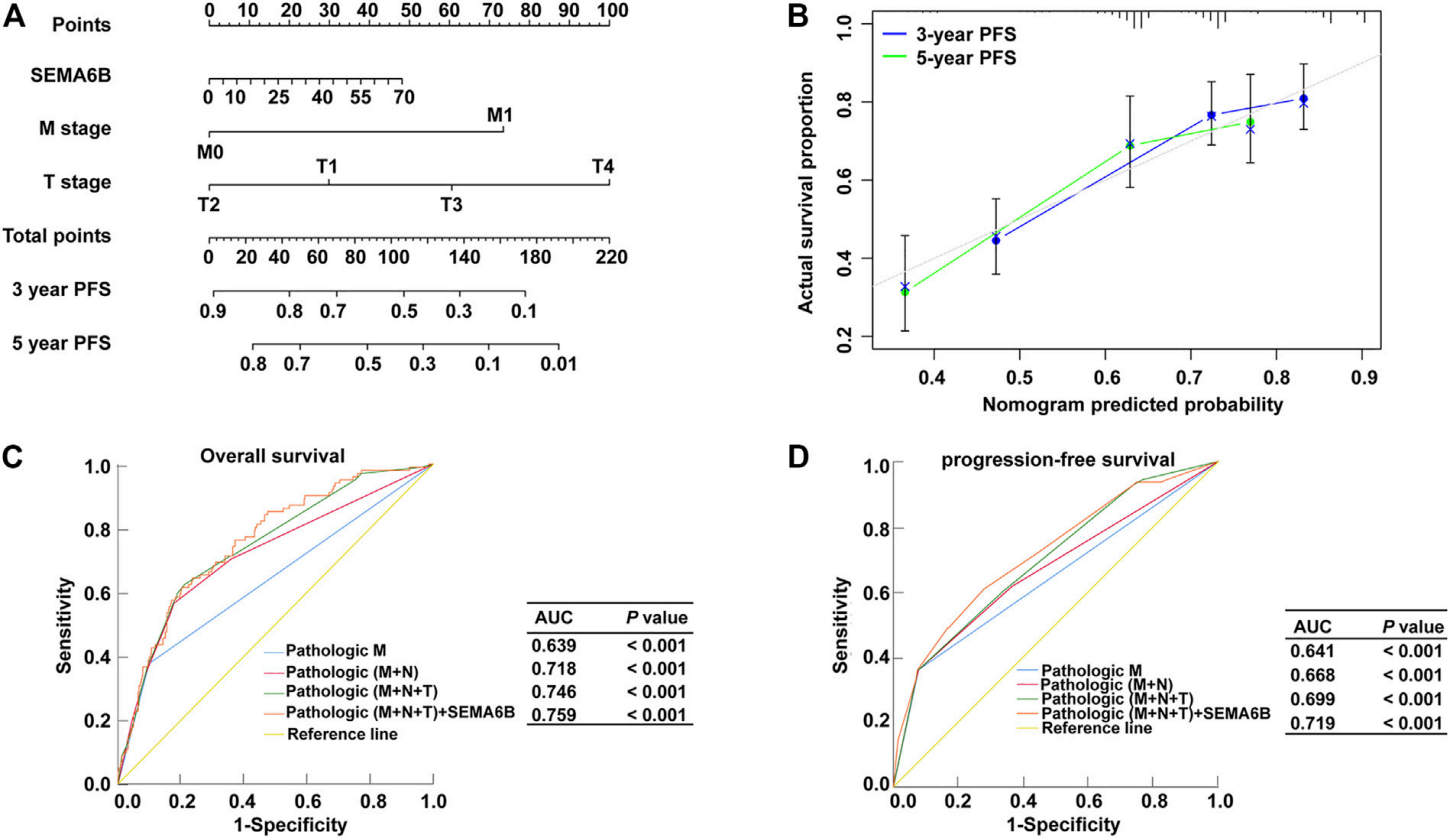
Nomogram model and ROC for survival prediction of CRC members. **(A)** Nomogram model to predict 3, and 5 years associated PFS probability. **(B)** The calibration curve of the nomogram when predicting 3- and 5 years PFS probability. ROC curves for predicting OS **(C)** and PFS **(D)** in CRC patients *via* prognostic models from the TCGA dataset. The *x*-axis indicates the false-positive rate, which is presented as ‘1-Specificity’. The *y*-axis indicates the true-positive rate, which is designated as “Sensitivity”.

According to the results of multivariate Cox regression analysis, the independent prognostic signature of SEMA6B, M stage, and N stage was enrolled to establish a nomogram model to predict the 3- and 5 year PFS probabilities of each patient for clinical practice (Figure 5A). The C-index of this model was 0.72 (95% CI, 0.70–0.75). Moreover, the calibration curve revealed that the 3- and 5 year PFS rates predicted by the nomogram were highly consistent with the actual observation outcomes (Figure 5B). These results demonstrated that the nomogram models show a favorable prognostic ability for predicting PFS. In addition, ROC analysis demonstrated that the AUC values for OS or PFS of the prediction model—including pathological M stage, N stage, T stage, and SEMA6B expression—were significantly improved from 0.639 to 0.759 for OS and from 0.641 to 0.719 for PFS (Figures 5C,D), indicating the additive predictive value of SEMA6B compared with that of known prognostic factors.

### SEMA6B-Related Biological Processes

**FIGURE 6:**
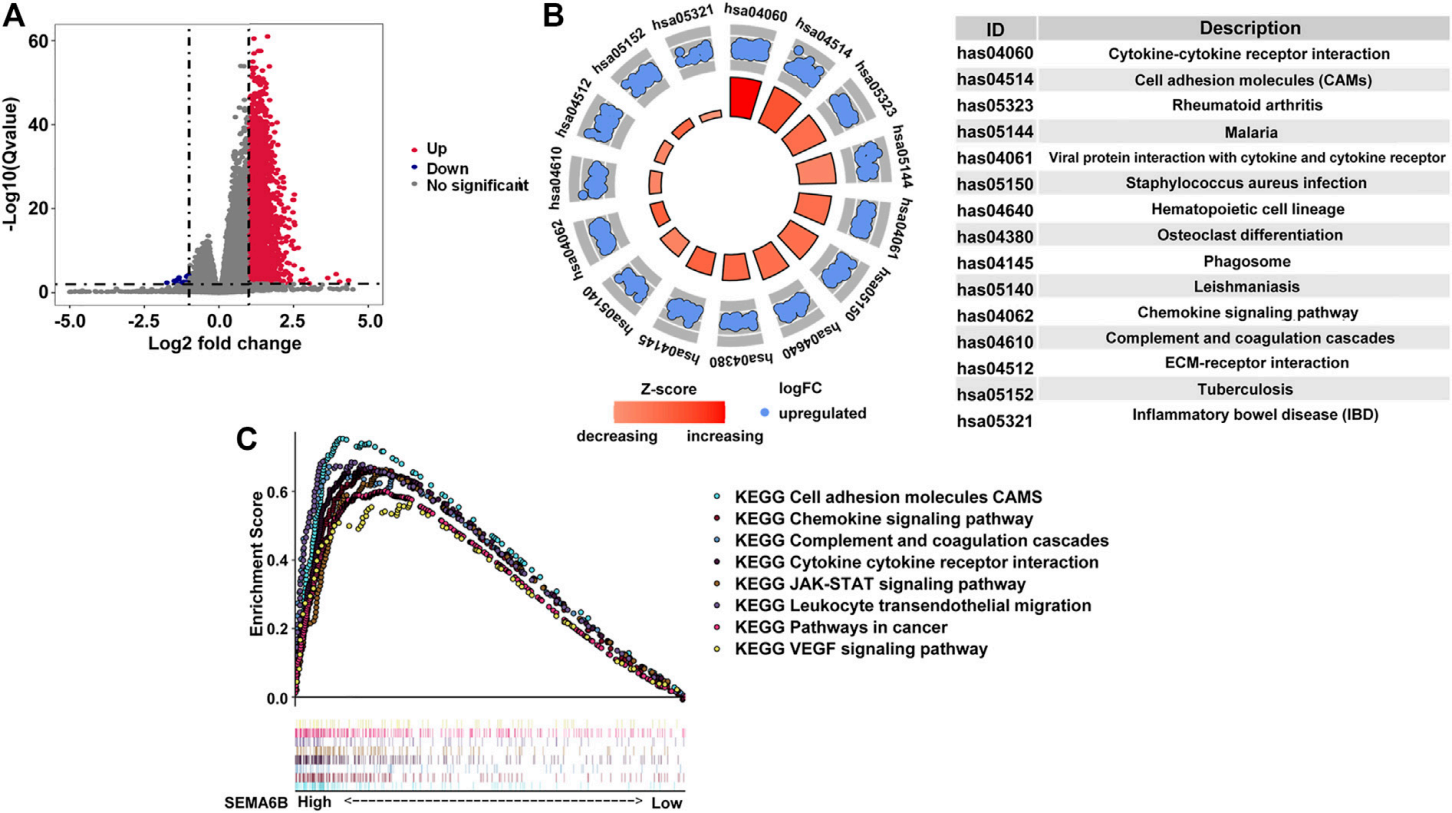
Identification differentially expressed genes (DEGs) in CRC patients from the TCGA dataset as well as KEGG and GSEA pathway enrichment analysis. **(A)** Volcano plot of aberrantly altered gene profiles between high and low SEMA6B expression groups. A total of 1,794 DEGs were identified. The red dots (n = 1,789) represent upregulated mRNAs, while the blue dots (n = 5) refer to downregulated mRNAs. The *x*-axis indicates the log2-fold change in gene expression, and the *y*-axis denotes the adjusted *p* values plotted in −Log10. **(B)** Top-15 KEGG pathways enriched by the overexpressed DEGs in the high SEMA6B expression group. *p* values were adjusted by the false-discovery rate (FDR) *via* R software. The size of each circle indicates the number of enriched genes, and the color denotes the adjusted *p* value. **(C)** GSEA enrichment plots of the TCGA-CRC dataset between high and low SEMA6B expression. A normalized enrichment score (NES) > 1 and adjusted *p* value (FDR) < 0.05 were used to determine significant gene sets.

**FIGURE 7:**
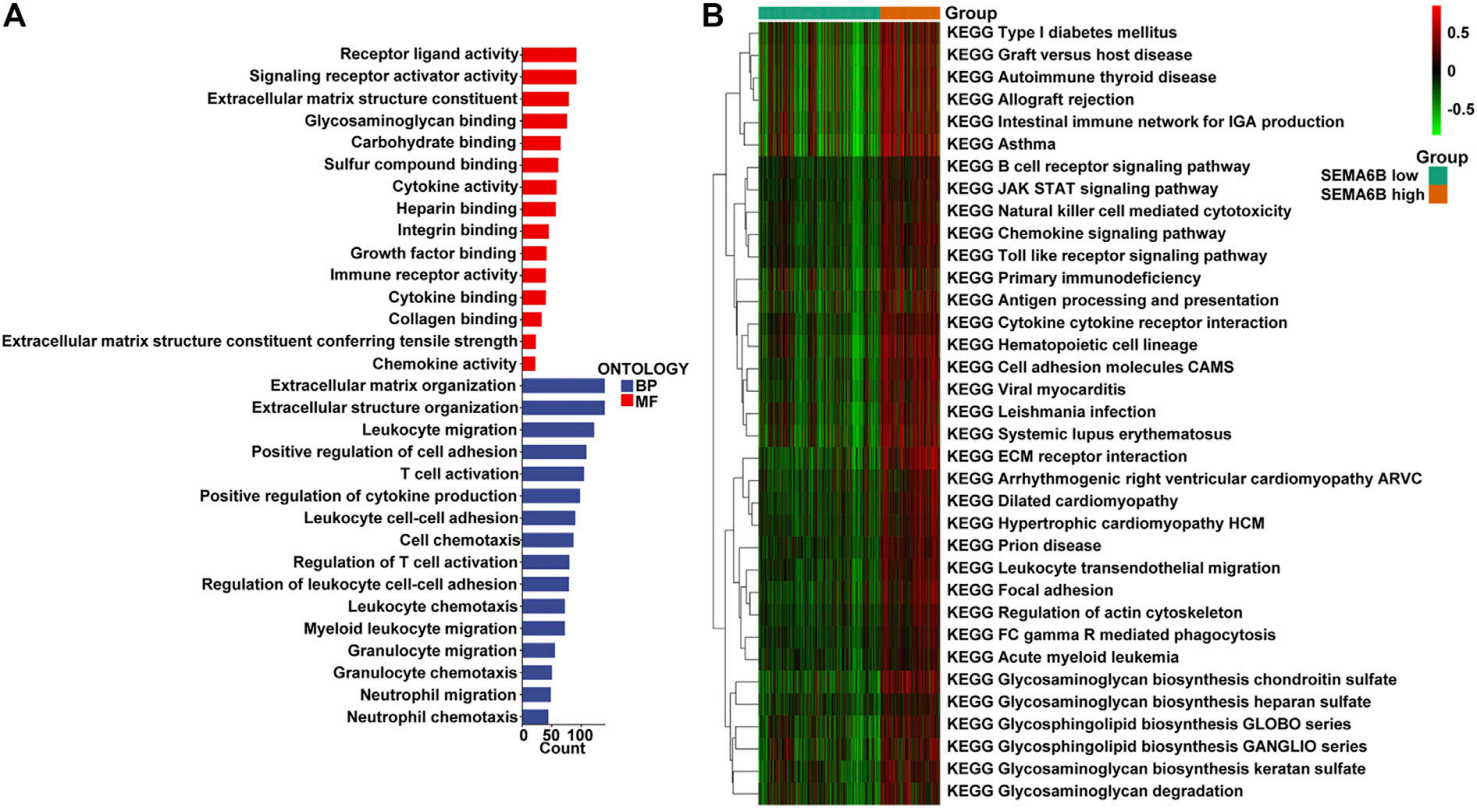
GO and GSVA enrichment analysis. **(A)** The 15-most significantly enriched GO terms in terms of upregulated mRNAs in the high SEMA6B expression group are listed according to their biological processes (BP) and molecular functions (MF). The length of each bar indicates the number of enriched genes. **(B)** The heatmap was used to visualize the result of GSVA enrichment analysis in high and low SEMA6B expression groups. The color changes from green to red, indicating an increase in the value of the enriched score. Brown represents the high SEMA6B group and green represents the low SEMA6B group.

According to the aforementioned results, SEMA6B may play an essential role in the biological functions of CRC. To clarify the biological roles of SEMA6B expression in CRC, DEGs were detected between high and low SEMA6B expression groups. Volcano plot analysis identified 1,794 DEGs (Figure 6A). Among them, 1,789 genes were upregulated and 5 genes were downregulated (Figure 6A). Then, the biological functions of these DEGs were explored by KEGG signaling pathway and GO annotation analysis. The top-15 pathways from the KEGG results showed that the most significantly altered pathways in the SEMA6B high expression group were those involving cytokine-cytokine receptor interactions, chemokine signaling pathways, rheumatoid arthritis, viral protein interactions with cytokines and cytokine receptors, and complement and coagulation cascades (Figure 6B). Furthermore, GSEA was conducted to determine the potential mechanism underlying SEMA6B in CRC. According to NES, we selected the most highly enriched signaling pathways. As shown in Figure 6C, the genes in the SEMA6B high expression group were mainly enriched in inflammatory activities and immune-related processes such as leukocyte transendothelial migration, chemokine signaling pathways, cytokine receptor interactions, and complement cascades. Furthermore, tumor-aggressiveness feature-related gene sets including those involving the Janus kinase-signal transducer and activator of transcription (JAK-STAT) signaling pathway, cancer pathways, and vascular endothelial growth factor (VEGF) signaling pathway were also significantly enriched in the high SEMA6B expression group. Meanwhile, the results of GO analysis revealed that many biological functions of these DEGs were primarily associated with inflammatory responses and adaptive immune responses from categories of biological processes and molecular functions (Figure 7A), respectively. To determine the biological behaviors based on distinct SEMA6B profiles in CRC patients, GSVA was performed. As expected, we found that the SEMA6B high-risk group was markedly enriched in tumor cell proliferation, immune response, EMT-related pathways, such as the JAK-STAT signaling pathway, antigen processing and presentation, natural killer cell mediated cytotoxicity, the chemokine signaling pathway, cytokine-cytokine receptor interaction, ECM receptor interaction, and focal adhesion (Figure 7B).

### SEMA6B Expression is Correlated With Immune Infiltration Levels

**FIGURE 8:**
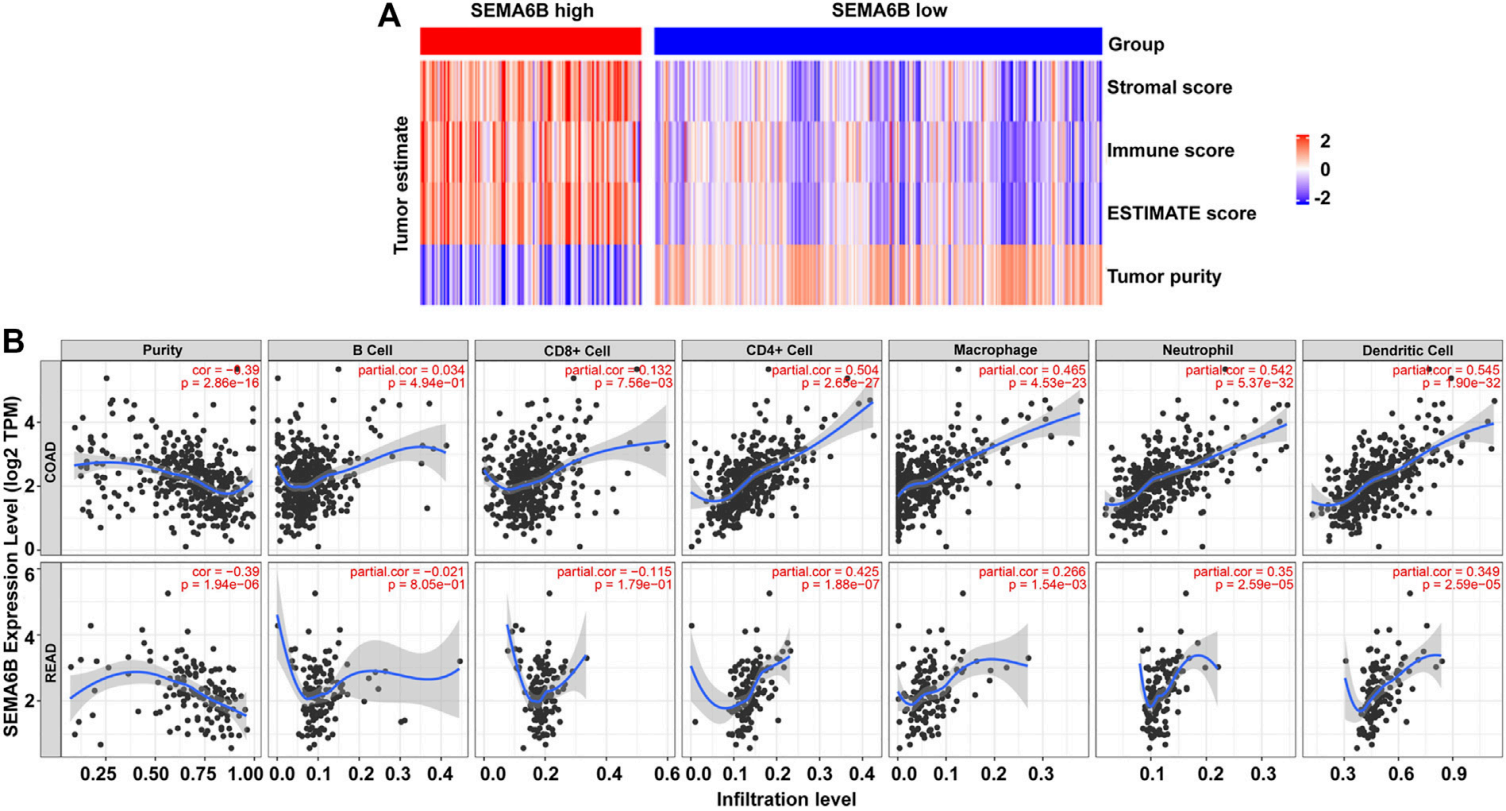
SEMA6B expression is correlated with immune infiltration in CRC patients. **(A)** Heatmaps of immune scores, stromal scores, ESTIMATE scores, and tumor purities for each patient between the high and low SEMA6B expression groups. **(B)** Correlations of SEMA6B expression with tumor purity and immune-cell infiltration levels in COAD and READ from the TIMER database.

Based on the ESTIMATE algorithm, patients’ stromal scores (range from −2634.54 to 1608.69), immune scores (range from −1272.49 to 2656.79), ESTIMATE scores (range from −3716.94 to 3710.24), and tumor purities (range from 0.409 to 0.998) are listed in Supplementary Table S1. As shown in Figure 8A, SEMA6B expression was positively correlated with stromal scores, immune scores, and ESTIMATE scores, while SEMA6B expression was negatively correlated with tumor purities.

Given that TILs are a prognostic indicator for CRC (Reissfelder et al., 2015; Berntsson et al., 2017), associations between SEMA6B gene expression and TILs infiltration levels in CRC were analyzed *via* the TIMER database. The results revealed that SEMA6B was negatively correlated with tumor purity in COAD (r = −0.39, *p* = 2.86E-16) and READ (r = −0.39, *p* = 1.94E-06), whereas it was strongly positively correlated with infiltrating levels of CD4^+^ T cells (r = 0.504, *p* = 2.65E-27), macrophages (r = 0.465, *p* = 4.53E-23), neutrophils (r = 0.542, *p* = 5.37E-32), and dendritic cells (r = 0.545, *p* = 1.90E-32) in COAD (Figure 8B). Similar trends in terms of correlations between SEMA6B and TILs infiltrating levels were also observed in READ (Figure 8B). Additionally, we also investigated the relationship between the expression levels of SEMA6B and immune infiltration in TISIDB database so as to further verify the role of SEMA6B in TME. Spearman’s correlation analysis illustrated that SEMA6B was strongly related to immune infiltration in COAD (Supplementary Figure S5A) and READ (Supplementary Figure S5B), especially for the five most significant infiltrators of immune cells, as follows: macrophages (r = 0.611 in COAD, r = 0.527 in READ), myeloid-derived suppressor cells (MDSCs) (r = 0.666 in COAD, r = 0.585 in READ), natural killer (NK) cells (r = 0.677 in COAD, r = 0.562 in READ), regulatory T cells (Tregs) (r = 0.619 in COAD, r = 0.586 in READ), and type-1 T helper (Th1) cells (r = 0.609 in COAD, r = 0.474 in READ).

To further determine correlations between SEMA6B and TILs, we analyzed relationships between SEMA6B and marker genes of different immune cells in COAD and READ *via* the TIMER and GEPIA databases. After correlation adjustments by purity, the findings demonstrated that expression levels of most marker sets of CD4^+^ T cells, Tregs, exhausted T cells, monocytes, tumor-associated macrophages (TAMs), M2 macrophages, and dendritic cells presented strong correlations with SEMA6B expression in COAD and READ (Table 1). In the GEPIA database, the analyses revealed that SEMA6B was strongly related to TILs infiltration in CRC tissues compared to that in normal colorectal samples, especially in terms of important signatures of pro-cancer immune cells, such as chemokine (C-C motif) ligand (CCL)-2, CD68, interleukin 10 (IL10) of TAMs, CD163, V-set and immunoglobulin domain-containing (VSIG4), membrane-spanning 4-domain subfamily A, member 4A (MS4A4A) of M2 macrophages, forkhead box protein P3 (FOXP3), transforming growth factor beta (TGFβ), C-C chemokine receptor 8 (CCR8), signal transducer and activator of transcription 5B (STAT5B) of Tregs, PD-1, CTLA4, and T-cell immunoglobulin mucin 3 (TIM-3) of T cell exhaustion (Table 2).

**TABLE 1:** Correlation analysis between SEMA6B and markers of immune cells in TIMER.

Description	Gene markers	COAD				READ			
		None		Purity		None		Purity	
		Cor	P	Cor	P	Cor	P	Cor	P
T cell	CD3D	0.412	***	0.321	***	0.295	***	0.199	***
	CD3E	0.505	***	0.420	***	0.387	***	0.299	**
	CD3G	0.391	***	0.311	***	0.285	***	0.181	0.030
	CD2	0.421	***	0.318	***	0.281	**	0.163	0.056
CD4^+^ T cell	CD4	0.621	***	0.573	***	0.572	***	0.520	***
CD8^+^ T cell	CD8A	0.418	***	0.354	***	0.317	***	0.196	0.020
	CD8B	0.224	***	0.189	**	0.094	0.229	0.058	0.498
	TBX21	0.515	***	0.439	***	0.393	***	0.326	***
	EOMES	0.420	***	0.366	***	0.243	0.001	0.105	0.219
	LCK	0.419	***	0.339	***	0.288	**	0.188	0.027
	IFNG	0.275	***	0.213	***	0.235	0.002	0.127	0.137
	PRF1	0.532	***	0.473	***	0.362	***	0.267	*
	GZMA	0.306	***	0.217	***	0.068	0.0387	-0.017	0.844
	GZMB	0.111	0.017	0.100	0.043	0.113	0.0148	0.057	0.509
	GZMH	0.438	***	0.381	***	0.258	**	0.133	0.118
	GZMK	0.422	***	0.351	***	0.235	0.002	0.075	0.381
	GZMM	0.482	***	0.414	***	0.377	***	0.322	**
	CXCL9	0.415	***	0.349	***	0.298	***	0.164	0.054
	CXCL10	0.363	***	0.300	***	0.262	**	0.131	0.123
Th1	IFN-γ(IFNG)	0.275	***	0.213	***	0.235	0.002	0.127	0.137
	TBX21	0.515	***	0.439	***	0.393	***	0.326	***
	TNF-α(TNF)	0.392	***	0.357	***	0.408	***	0.345	***
	STAT4	0.392	***	0.303	***	0.366	***	0.260	*
	STAT1	0.393	***	0.352	***	0.238	*	0.087	0.308
Th2	STAT5A	0.411	***	0.388	***	0.248	*	0.220	*
	IL13	0.284	***	0.235	***	0.234	*	0.129	0.129
	STAT6	0.194	***	0.203	***	0.176	0.023	0.257	*
	GATA3	0.557	***	0.509	***	0.486	***	0.413	***
Tfh	CXCR5	0.490	***	0.407	***	0.299	***	0.190	0.025
	CXCL3	−0.029	0.540	-0.039	0.432	0.019	0.809	0.047	0.583
	BCL6	0.607	***	0.548	***	0.394	***	0.368	***
	IL21	0.241	***	0.212	***	0.018	0.814	−0.066	0.441
Th17	IL17A	−0.089	0.056	−0.096	0.053	−0.089	0.253	−0.044	0.607
	RORC	−0.079	0.093	−0.069	0.165	−0.160	0.039	−0.144	0.092
	IL23A	0.134	*	0.114	0.022	0.025	0.746	−0.040	0.643
	STAT3	0.408	***	0.367	***	0.323	***	0.274	*
Treg	FOXP3	0.617	***	0.565	***	0.545	***	0.499	***
	IKZF2	0.253	***	0.197	***	0.141	0.070	0.040	0.643
	IL10	0.498	***	0.474	***	0.402	***	0.323	**
	TGFβ(TGFB1)	0.704	***	0.640	***	0.653	***	0.611	***
	CCR8	0.522	***	0.480	***	0.443	***	0.345	***
	STAT5B	0.297	***	0.308	***	0.371	***	0.334	***
T cell exhaustion	PD-1(PDCD1)	0.516	***	0.449	***	0.426	***	0.349	***
	GZMB	0.111	0.017	0.100	0.043	0.113	0.0148	0.057	0.509
	LAG3	0.517	***	0.452	***	0.453	***	0.418	***
	CTLA4	0.529	***	0.457	***	0.418	***	0.312	**
	TIM-3(HAVCR2)	0.586	***	0.526	***	0.472	***	0.387	***
B cell	CD19	0.381	***	0.281	***	0.141	0.070	0.076	0.376
	CD79A	0.433	***	0.343	***	0.236	*	0.119	0.163
Monocyte	CD86	0.600	***	0.536	***	0.451	***	0.353	***
	CD115(CSF1R)	0.725	***	0.705	***	0.672	***	0.667	***
TAM	CCL2	0.566	***	0.525	***	0.550	***	0.466	***
	CD68	0.590	***	0.547	***	0.426	***	0.386	***
	IL10	0.498	***	0.474	***	0.402	***	0.323	**
M1 Macrophage	INOS(NOS2)	0.065	0.166	0.027	0.589	0.040	0.606	0.076	0.371
	IRF5	0.260	***	0.280	***	0.325	***	0.317	**
	COX2(PTGS2)	0.355	***	0.308	***	0.421	***	0.328	***
M2 Macrophage	CD163	0.677	***	0.645	***	0.636	***	0.595	***
	VSIG4	0.635	***	0.592	***	0.520	***	0.467	***
	MS4A4A	0.572	***	0.515	***	0.520	***	0.450	***
Neutrophils	CD66b (CEACAM8)	−0.093	0.468	−0.068	0.171	−0.005	0.948	0.116	0.073
	CCR7	0.542	***	0.470	***	0.389	***	0.335	***
	CD11b (ITGAM)	0.717	***	0.686	***	0.580	***	0.532	***
Natural killer cell	KIR3DL1	0.294	***	0.226	***	0.219	*	0.176	0.038
	KIR3DL2	0.299	***	0.260	***	0.248	*	0.172	0.043
	KIR3DL3	0.124	*	0.114	0.021	−0.065	0.403	-0.122	0.152
	KIR2DS4	0.205	***	0.194	***	0.153	0.050	0.058	0.500
	KIR2DL1	0.273	***	0.243	***	0.066	0.398	−0.017	0.847
	KIR2DL3	0.237	***	0.194	***	0.151	0.051	0.109	0.200
	KIR2DL4	0.284	***	0.223	***	0.169	0.030	0.036	0.673
Dendritic cell	BDCA-1(CD1C)	0.358	***	0.288	***	0.274	**	0.168	0.048
	BDCA-4(NRP1)	0.725	***	0.680	***	0.586	***	0.485	***
	CD11c (ITGAX)	0.742	***	0.695	***	0.640	***	0.590	***
	HLA-DPB1	0.640	***	0.582	***	0.515	***	0.458	***
	HLA-DQB1	0.387	***	0.316	***	0.340	***	0.242	*
	HLA-DRA	0.489	***	0.415	***	0.370	***	0.231	*
	HLA-DPA1	0.536	***	0.470	***	0.404	***	0.274	*

COAD, colon adenocarcinoma; READ, rectal adenocarcinoma; TAM, tumor-associated macrophage; Th, T helper cell; Tfh, Follicular helper T cell; Treg, regulatory T cell; Cor, R value of Spearman’s correlation; None, correlation without purity adjustment. Purity, correlation adjusted by purity. *p* < 0.01 *, *p* < 0.001 **, *p* < 0.0001 ***.

**TABLE 2:** Correlation analysis between SEMA6B expression and related genes and markers of immune cells in normal tissues and CRC tissues by GEPIA.

Description	Gene markers	COAD + READ			
		Normal		Tumor	
		Cor	P	Cor	P
Monocyte	CD86	0.098	0.500	0.670	***
	CD115(CSF1R)	0.290	0.036	0.800	***
TAM	CCL2	0.100	0.480	0.710	***
	CD68	0.120	0.420	0.500	***
	IL10	0.027	0.850	0.630	***
M1 Macrophage	IRF5	0.085	0.56	0.210	***
	INOS(NOS2)	−0.003	0.980	-0.007	0.890
	COX2(PTGS2)	0.160	0.280	0.230	***
M2 Macrophage	VSIG4	0.310	0.025	0.670	***
	CD163	0.400	*	0.740	***
	MS4A4A	0.160	0.270	0.690	***
T cell	CD3D	−0.140	0.320	0.350	***
	CD3E	−0.029	0.840	0.430	***
	CD3G	−0.100	0.490	0.360	***
	CD2	−0.083	0.560	0.400	***
CD4^+^ T cell	CD4	0.270	0.055	0.650	***
CD8^+^ T cell	CD8A	−0.019	0.890	0.280	***
	CD8B	−0.110	0.460	0.150	**
	TBX21	−0.039	0.780	0.390	***
	EOMES	−0.088	0.540	0.320	***
	LCK	−0.051	0.720	0.160	**
	IFNG	−0.099	0.490	0.210	***
	PRF1	-0.001	1.000	0.190	**
	GZMA	−0.240	0.089	0.200	***
	GZMB	−0.052	0.720	−0.061	0.250
	GZMH	−0.110	0.450	0.250	***
	GZMK	−0.220	0.120	0.380	***
	GZMM	−0.044	0.760	0.360	***
	CXCL9	−0.120	0.400	0.310	***
	CXCL10	−0.130	0.350	0.160	***
Th1	IFN-γ(IFNG)	−0.099	0.490	0.210	***
	TBX21	−0.039	0.780	0.390	***
	TNF-α(TNF)	0.320	0.023	0.330	***
	STAT4	0.026	0.860	0.520	***
	STAT1	−0.094	0.510	0.280	***
Th2	IL13	0.048	0.740	0.220	***
	STAT6	0.290	0.036	0.057	0.280
	GATA3	−0.030	0.840	0.400	***
	STAT5A	0.320	0.022	0.400	***
Tfh	CXCR5	−0.049	0.740	0.190	**
	CXCL3	0.170	0.240	−0.042	0.420
	BCL6	0.340	0.016	0.720	***
	IL21	-−0.081	0.570	0.260	***
Th17	IL17A	0.042	0.770	−0.076	0.140
	RORC	0.017	0.910	−0.019	0.720
	IL23A	0.390	*	0.087	0.098
	STAT3	0.390	*	0.470	***
Treg	FOXP3	0.120	0.410	0.510	***
	IKZF2	−0.006	0.970	0.390	***
	IL10	0.027	0.850	0.630	***
	TGFβ(TGFB1)	0.540	***	0.640	***
	CCR8	0.170	0.240	0.500	***
	STAT5B	0.290	0.037	0.430	***
T cell exhaustion	LAG3	0.036	0.800	0.180	**
	CTLA4	0.110	0.420	0.340	***
	TIM-3(HAVCR2)	0.170	0.240	0.620	***
	PD-1(PDCD1)	0.079	0.580	0.330	***
	GZMB	−0.052	0.720	−0.061	0.250
B cell	CD79A	−0.040	0.780	0.340	***
	CD19	−0.098	0.490	0.310	***
Neutrophils	CD66b (CEACAM8)	0.071	0.620	−0.026	0.620
	CD11b (ITGAM)	0.290	0.038	0.720	***
	CCR7	0.082	0.570	0.410	***
Natural killer cell	KIR3DL1	−0.110	0.460	0.140	*
	KIR3DL2	0.078	0.590	0.350	***
	KIR3DL3	0.190	0.190	0.240	***
	KIR2DS4	0.180	0.200	0.140	*
	KIR2DL1	0.180	0.220	0.260	***
	KIR2DL3	−0.096	0.500	0.250	***
	KIR2DL4	−0.220	0.120	0.230	***
Dendritic cell	BDCA-1(CD1C)	-−0.150	0.310	0.330	***
	BDCA-4(NRP1)	0.560	***	0.870	***
	CD11c (ITGAX)	0.140	0.340	0.640	***
	HLA-DQB1	0.042	0.770	0.290	***
	HLA-DRA	−0.150	0.290	0.450	***
	HLA-DPB1	0.046	0.750	0.480	***
	HLA-DPA1	0.007	0.960	0.410	***

COAD, colon adenocarcinoma; READ, rectal adenocarcinoma; TAM, tumor-associated macrophage; Th, T helper cell; Tfh, Follicular helper T cell; Treg, regulatory T cell; Cor, R value of Spearman’s correlation; tumor, correlation analysis in tumor tissues from TCGA; normal, correlation analysis in normal tissues from TCGA. *p* < 0.01 *, *p* < 0.001 **, *p* < 0.0001 ***.

### SEMA6B Overexpression is Indicative of the Tumor Immunosuppressive Microenvironment

**FIGURE 9:**
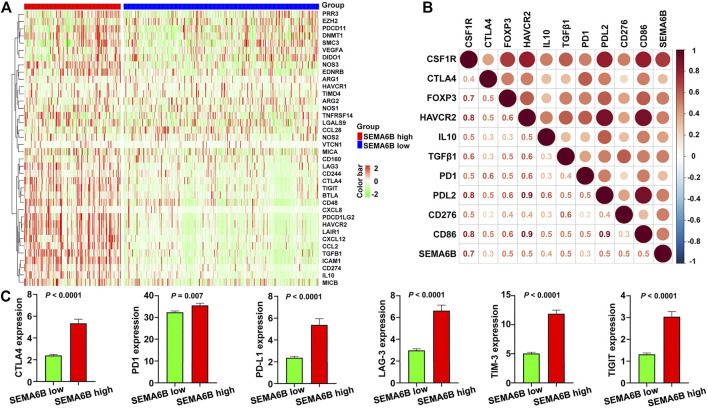
High SEMA6B expression indicates the tumor immunosuppressive microenvironment. **(A)** Heatmap of the gene profiles involved in the negative regulation of the cancer-immunity cycle in the high and low SEMA6B expression groups using the TCGA-CRC cohort. **(B)** Heatmap displaying correlations between SEMA6B expression and immunosuppressive genes. The number in each small box indicates the Pearson correlation coefficient between the two corresponding genes. **(C)** Comparisons of the expression levels of representative immune checkpoint genes in the high and low SEMA6B expression groups.

To further determine the influence of SEMA6B on the tumor microenvironment, we investigated correlations between SEMA6B and expression of genes negatively regulating the cancer-immunity cycle, which consists of a cycle of processes involving eradication of cancer by the immune system (Chen and Mellman 2013). Gene signatures were downloaded from the Tracking Tumor Immunophenotype website (http://biocc.hrbmu.edu.cn/TIP/index.jsp) (Xu et al., 2018). Unsupervised hierarchical clustering analyses revealed that genes involved in the negative regulation of the cancer-immunity cycle were mostly upregulated in the high SEMA6B expression group (Figure 9A). A correlation coefficient heatmap showed that SEMA6B had a significant positive correlation with immunosuppressive molecules, including colony stimulating factor 1 receptor (CSF1R), PD-L2, FOXP3, CD86, and TGFβ1 (Figure 9B). As expected, the expression levels of immune checkpoints—such as PD-L1, PD-1, CTLA-4, T cell immunoreceptor with Ig and ITIM domains (TIGIT), TIM-3, and lymphocyte activation gene 3 (LAG-3)—in the high SEMA6B expression group were significantly higher than those in the low SEMA6B expression group (Figure 9C). These results suggest that SEMA6B plays a vital role in immune escape *via* promoting the tumor immunosuppressive microenvironment.

### SEMA6B Knockdown Blocks Cell Proliferation, Migration, Invasion, and the mRNA Expression of Immunosuppressive Molecules in Colon Cancer Cells

**FIGURE 10:**
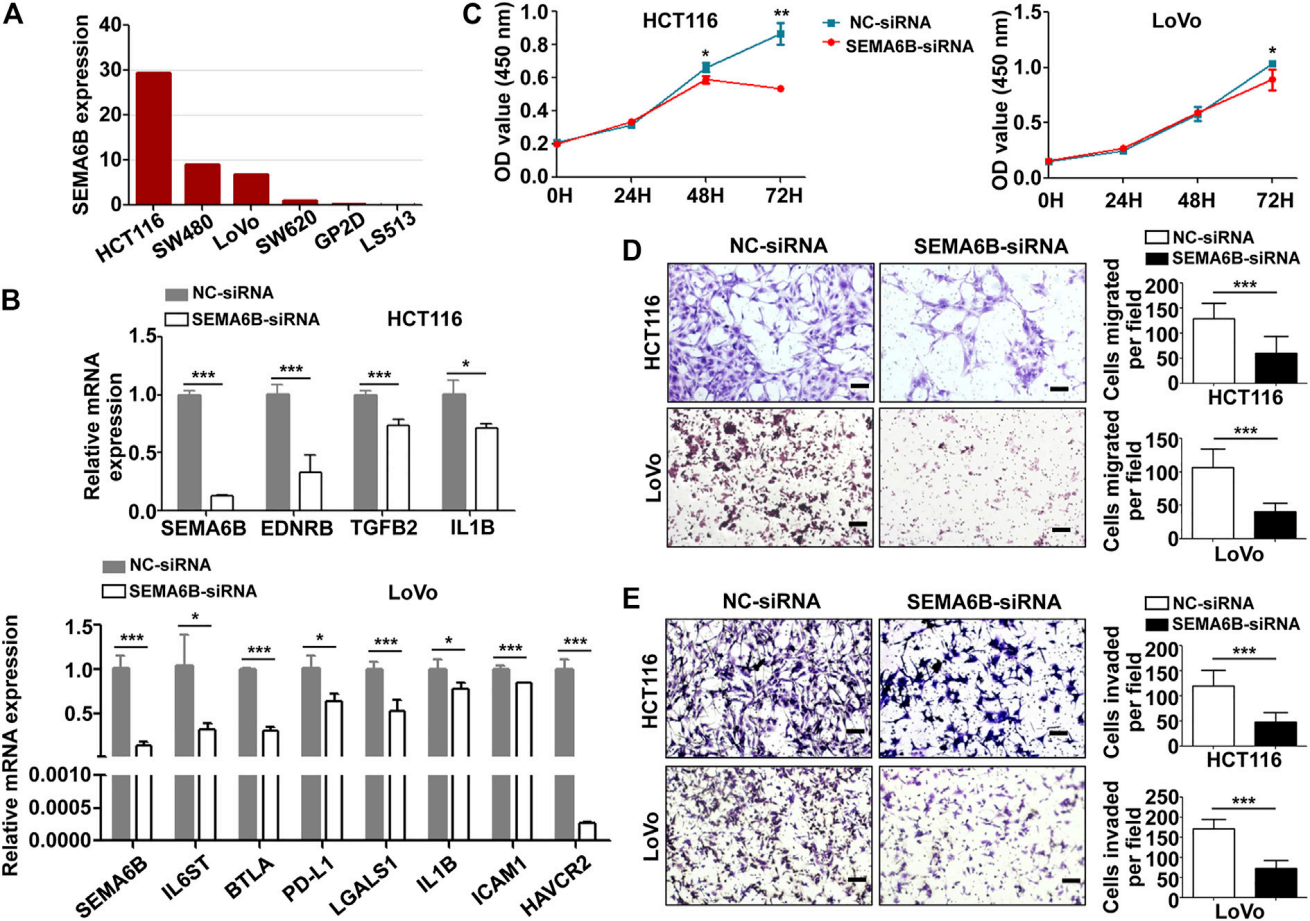
SEMA6B knockdown retards the malignant biological behavior of colon cancer cells and the expression of immunosuppressive molecules. HCT116 and LoVo cells were transfected with NC-siRNA or SEMA6B-siRNA. **(A)** The mRNA expression level in different colon cancer cells from the CCLE website. **(B)** QRT-PCR analysis was performed to examine the knockdown efficiency of SEMA6B and the relative mRNA expression of immunosuppressive genes. **(C)** CCK-8 assay was applied to evaluate cell viability. Transwell assay was employed to count the number of migrated **(D)** and invaded **(E)** cells. *p < 0.05, **p < 0.01, and ***p < 0.001.

The mRNA level of SEMA6B in different colon cancer cell lines was investigated through CCLE datasets. Bar plots show that HCT116, SW480, LoVo, SW620, GP2D, and LS513 are the top six colon cancer cell lines with the highest SEMA6B expression levels (Figure 10A). Therefore, HCT116 and LoVo cell lines were chosen for further study. QRT-PCR analysis revealed that SEMA6B silencing can not only significantly reduce the expression of SEMA6B, but also decrease the mRNA levels of immunosuppressive molecules, EDNRB, TGFB2, IL1B, IL6ST, BTLA, PD-L1, LGALS1, ICAM1, HAVCR2 in the selected cells lines compared to the corresponding controls (Figure 10B). Knockdown of SEMA6B significantly reduced the proliferation in both cell lines according to the results of the CCK-8 assay (Figure 10C). Furthermore, the cell numbers that migrated through the membrane were significantly reduced in the SEMA6B-silenced groups of both cell lines according to the results of the Transwell migration assay (Figure 10D). Transwell invasion assay also showed that SEMA6B silencing significantly decreased the degree of invasiveness in both selected colon cancer cell lines (Figure 10E). These data demonstrate that SEMA6B knockdown reduces the growth and progression of colon cancer cells as well as suppresses the formation of an immunosuppressive microenvironment.

## Dicussion

Accumulating evidence has indicated that the intra-tumoral immune contexture (i.e., type, functional orientation, density, and location of immune cells) of solid tumors may be a dominant determinant of clinical outcomes (Becht et al., 2016; Fridman et al., 2017). Despite CRC being one of the major cancer types for which new immune-based cancer treatments are currently in development, the prognosis of patients with advanced CRC remains poor (Emambux and Tachon 2018; Ciardiello and Vitiello 2019). Therefore, it has been necessary to further identify immune-related biomarkers and better elucidate the underlying molecular mechanisms of CRC to improve patient prognosis and guide the development of immunomodulation for CRC treatments. In the present study, we focused on SEMA6B, a member of the semaphorin axon-guidance family, which exhibits immune functions related to the control of cellular movements and cell-cell communication (Tawarayama et al., 2010; Koivisto et al., 2020). Given that our knowledge of the role of SEMA6B in cancers is limited, in the present study, we aimed at investigating its prognostic value in CRC and revealed its associated biological functions by performing a comprehensive analysis of population databases.

Here, we found that the mRNA expression levels of SEMA6B were significantly increased in CRC compared with those in normal colorectal tissues using the TCGA and GEO databases. A similar trend in the protein expression levels of SEMA6B was also observed in the HPA database. In addition, our data demonstrated that gene hypomethylation was influential in upregulation of SEMA6B expression in CRC. These findings imply that enhanced SEMA6B expression had considerable effects on CRC progression in a manner that may be due to epigenetic alterations. Of note, SEMA6B levels were related with the disease type, venous invasion, T stage, MSI, KRAS mutation, and CMS of CRC patients. Moreover, high SEMA6B expression was found to predict worse survival in all cohorts, and was further shown to be an independent prognostic factor of PFS in CRC patients. The nomogram based on SEMA6B, M stage, and N stage could facilitate accurate prediction of the 3- and 5 year PFS probabilities in CRC patients. To our knowledge, this is the first study to report a consistent association between increased SEMA6B levels and poor prognosis in CRC. These results indicate a malignant biological influence of high SEMA6B levels in CRC.

Studies based on the roles of SEMA6B have also been reported for other malignant human tumors. In breast cancer, high levels of SEMA6B in human MCF-7 cells exhibit an *in vitro* invasive capacity, and show potential as a key regulator of tumor progression (Murad and Collet, 2006). Recently, SEMA6B has been shown to exhibit higher expression levels in testicular cancer tissues than in normal tissues and is considered to be a predictor of poor prognosis in patients with testicular germ-cell tumors (Ji and Wang, 2020). On the contrary, SEMA6B has been reported to be downregulated in human glioblastoma cells upon prolonged treatment with the anti-tumor action of all-trans retinoic acid (ATRA) (Correa et al., 2001). Increasing evidence has indicated that SEMA6B is related to macrophages and correlates with a favorable prognosis in glioma patients (Sun et al., 2019). Thus, these conflicting findings suggest that SEMA6B may play differential roles in different kinds of human cancers, and that discrepancies between SEMA6B expression and prognostic values may derive from underlying mechanisms pertinent to specific biological properties in various cancers. Exploring and revealing the mechanisms of SEMA6B in CRC may facilitate the discovery of novel therapeutic approaches for CRC patients.

Biological pathway analysis and functional enrichment analysis in our present study illustrated that immune-related processes, inflammatory activities, and cancer signaling pathways were significantly enriched in the high SEMA6B expression group. Previous studies have indicated that immune infiltrating levels in tumor sites influence prognosis and the response rate of chemotherapy and immunotherapy in CRC patients (Waniczek et al., 2017). The ESTIMATE algorithm was first reported by Yoshihara et al. to assess immune-cell infiltration and the presence of stromal cells based on gene expression data (Yoshihara and Shahmoradgoli, 2013). In the present study, we revealed that high SEMA6B expression was positively associated with higher stromal scores, immune scores, and ESTIMATE scores but was negatively association with tumor purity. Another noteworthy finding of the present study was that SEMA6B expression was correlated with diverse immune infiltration levels in CRC. Our results showed that there were moderate-to-strong positive relationships between SEMA6B expression levels and infiltration levels of macrophages, MDSCs, NK cells, Tregs, and Th1 cells, as well as significantly positive correlations between infiltrating levels of CD4^+^ T cells, neutrophils, and dendric cells and SEMA6B expression in CRC. Likewise, the relationships between gene markers of different immune cells and SEMA6B expression are suggestive of a pivotal role of SEMA6B in regulating the tumor immune microenvironment. In addition to identifying markers of CD8^+^ T-cell activation, we also found that NK cells were more active in tumors with high SEMA6B expression, which indicates that these tumors may have an antitumor immune microenvironment (Qu et al., 2018). However, the above-mentioned immune cells do not serve as the key effectors that destroy tumor cells in CRC patients. This phenomenon may be due to the following possibilities. First, immunosuppressive cells such as Tregs, M2 macrophages, and MDSCs (Qu et al., 2018; Xiang and Gilkes 2019) are known to dominate the immune microenvironment in tumors with high SEMA6B expression. Second, SEMA6B has the potential to induce CD8^+^ T-cell exhaustion, as reflected by positive correlations with T-cell exhaustion markers. TIM-3, a crucial surface protein on exhausted T cells (Anderson 2012), was highly correlated with SEMA6B expression in CRC in the present study. Third, SEMA6B expression represents weak correlations with gene markers of M1 macrophages, whereas M2 macrophage indicators exhibited strong correlations in the present study. Previous studies have underscored the dualistic roles of macrophages in tumors: the M1 phenotype exhibits proinflammatory and tumoricidal activities, whereas the M2 phenotype exhibits anti-inflammatory activities and exerts functions in immunosuppression and malignant progression of tumors (Biswas and Mantovani 2010; Mantovani and Sica 2010). Our present results suggest a potential regulatory role of SEMA6B in polarization of TAMs with the M2 phenotype for evading immune surveillance.

The cancer-immunity cycle is a series of functional stepwise events required to obtain an efficient control of cancer growth by the immune system (Chen and Mellman 2013; Horton et al., 2019). This process consists of the following seven steps (Chen and Mellman 2013): 1) releasing of cancer cell antigens; 2) cancer-antigen presentation; 3) priming and activation; 4) trafficking of immune cells to tumors; 5) infiltration of immune cells into tumors; 6) recognition of cancer cells by T cells; and 7) killing of cancer cells. Immune checkpoint molecules—such as PD-1, PD-L1, and CTLA4—can inhibit the development of an active immune response by acting primarily at the level of T-cell development and proliferation (step 3) (Chen and Mellman 2013; Chen and Mellman 2017). Negative gene sets in the cancer-immunity cycle—including PD-1, PD-L1, LAG-3, TIM-3, and TIGIT—can have an inhibitory function that primarily acts to modulate active immune responses in the tumor bed (step 7) (Chen and Mellman 2013; Chen and Mellman 2017). Therefore, tracking tumor immunophenotypes may be essential for further elucidating the mechanisms of cancer immunity and progressing the development of biomarkers of responses to immunotherapy. Our present study demonstrated that in CRC patients with high SEMA6B expression, the genes involved in the prevention of T-cell priming and the induction of tolerance were increased, and immune checkpoints—such as PD1, PD-L1, CTLA-4, LAG3, TIM-3, and TIGIT—were also upregulated in this group. Hence, accumulating evidence suggests that SEMA6B may contribute to tumor development by both attenuation of tumor-specific cytotoxic T-cell responses and induction of an immunosuppressive state ( Kigel and Rabinowicz 2011; Ge and Li 2013; Cui and Bian 2021 ).

We conducted SEMA6B knockdown experiments to further confirm the biological role of SEMA6B in two colon cancer cell lines, i.e., HCT116 and LoVo. The results demonstrate that SEMA6B silencing enables a reduction in proliferation, migration and invasion *in vitro*; meanwhile, the mRNA expression levels of immunosuppressive molecules were also diminished in silenced colon cancer cells by qRT-PCR. Gui et al. reported that siRNA-mediated knockdown of SEMA6B weakened gallbladder cancer cell invasion and migration (Cui and Bian 2021). Ge et al. also discovered that SEMA6B may promote gastric cancer invasion and metastasis and represents a reliable biomarker for the clinical diagnosis and therapy of gastric cancer (Ge and Li 2013). Overexpression of SEMA6B in BHK-21 cells could promote cell proliferation, and the inhibition of SEMA6B signaling suppresses tumor formation for the sake of abrogation of bFGF and VEGF signaling (Kigel and Rabinowicz 2011). Overall, our findings reveal that SEMA6B may play a key role in regulating CRC progression and helps shape the immunosuppressive microenvironment. However, further studies are required to validate the biological functions of SEMA6B in CRC.

There were some limitations of our present study. First, only transcriptomic expression of SEMA6B expression with clinical data was analyzed to predict prognosis in CRC from public databases; thus, our results should be validated in larger sample size. Second, this was a retrospective study with selection biases inherent in the cohorts; thus a prospective study is also needed. Third, the molecular mechanisms of SEMA6B in CCR remain unclear, despite a series of functional annotations and enrichment analysis being investigated. Thus, further study is required to recognize the potential biological mechanisms of SEMA6B using additional experimental approaches.

In conclusion, our present study explored the clinical value and biological processes of SEMA6B, using CRC samples from the TCGA and GEO databases on a large scale. Our data revealed that high SEMA6B expression was significantly correlated with cancer progression, poor survival, and immune infiltration in patients with CRC. Moreover, high SEMA6B expression was correlated with increased immunosuppressive cellular infiltration and the expression of immune checkpoints. Moreover, *in vitro* cell studies validate that overexpress SEMA6B may promote proliferation and metastasis in two colon cancer cell lines, and help to foster an immunosuppressive microenvironment. Our present findings therefore offer novel insights into how SEMA6B affects prognosis and the immune microenvironment in CRC and suggests that SEMA6B may serve as a potential biomarker for developing immunotherapeutic strategies for assessing the efficacy and responsiveness of CRC treatments.

## Data Availability

The datasets presented in this study can be found in online repositories. The names of the repository/repositories and accession number(s) can be found in the article/Supplementary Material.
