# Supplementary material for: SEMA6B Overexpression Predicts Poor Prognosis and Correlates With the Tumor Immunosuppressive Microenvironment in Colorectal Cancer
Source: Front Mol Biosci. 2021 Dec 6;8:687319. doi: 10.3389/fmolb.2021.687319 (PMC8687481; doi:10.3389/fmolb.2021.687319)
Supplement: Supplementary file 1 [file DataSheet1.DOCX]

**
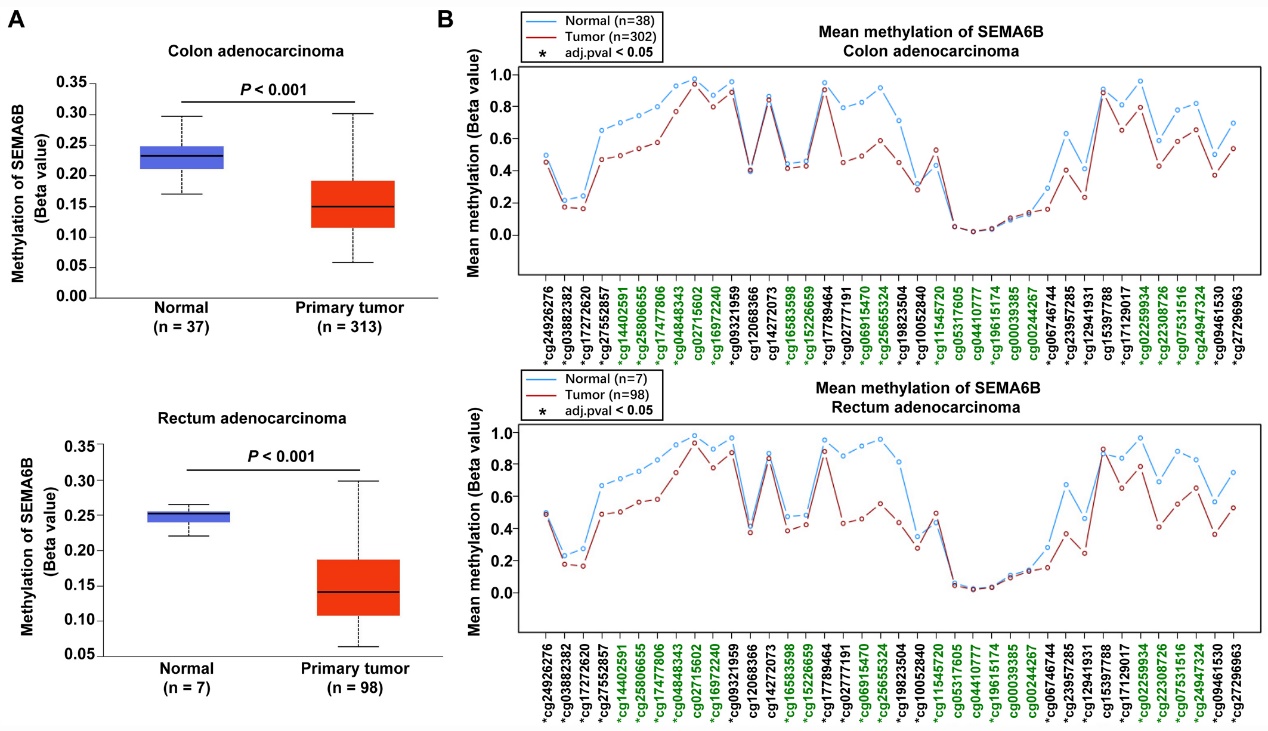
**

**Supplementary Figure S1** SEMA6B DNA methylation in CRC tissues and normal tissues. **(A)** Differences in methylation levels of SEMA6B between CRC tissues and adjacent normal tissues from the UALCAN database. **(B)** SEMA6B methylation in CRC samples compared with that in normal samples from the Wanderer database. Green font represents CpG islands; the adj.pval represents adjusted *P* values and * represents adjusted *P* values < 0.05.


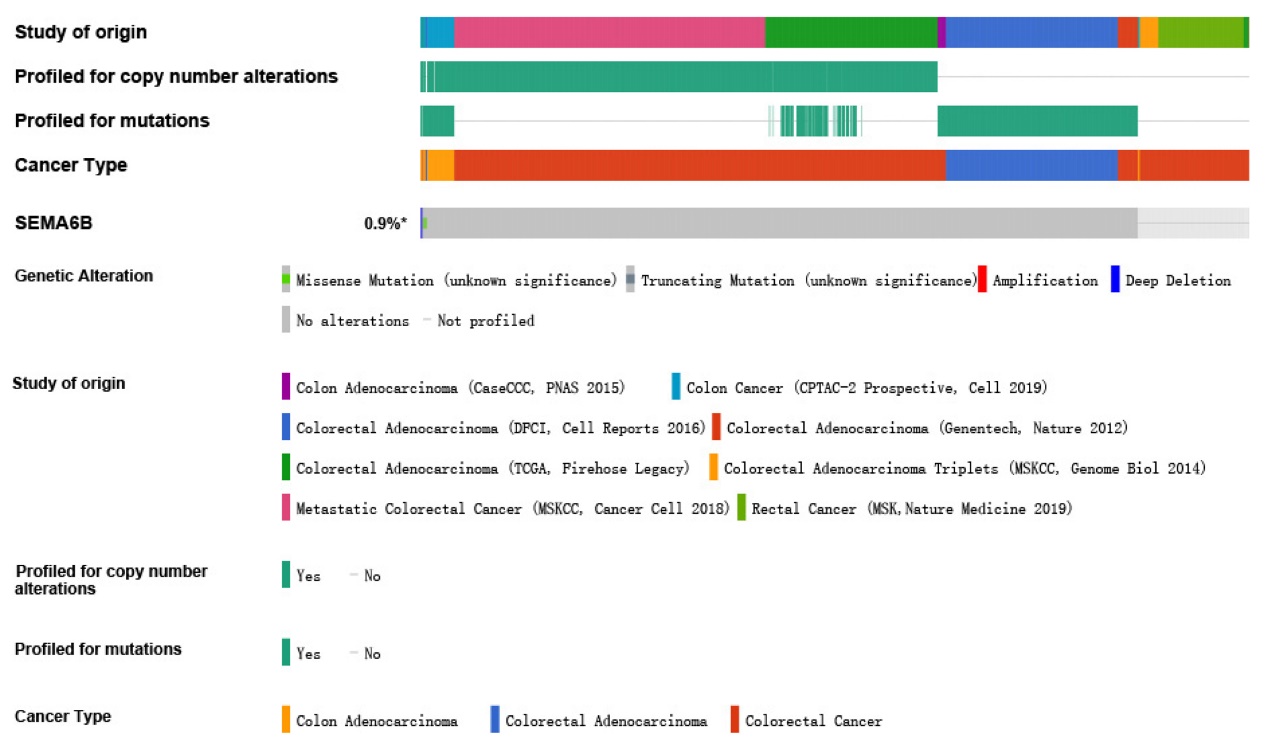


**Supplementary Figure S2** Status of somatic mutations and SEMA6B gene mutations in CRC samples using the cBioPortal database.


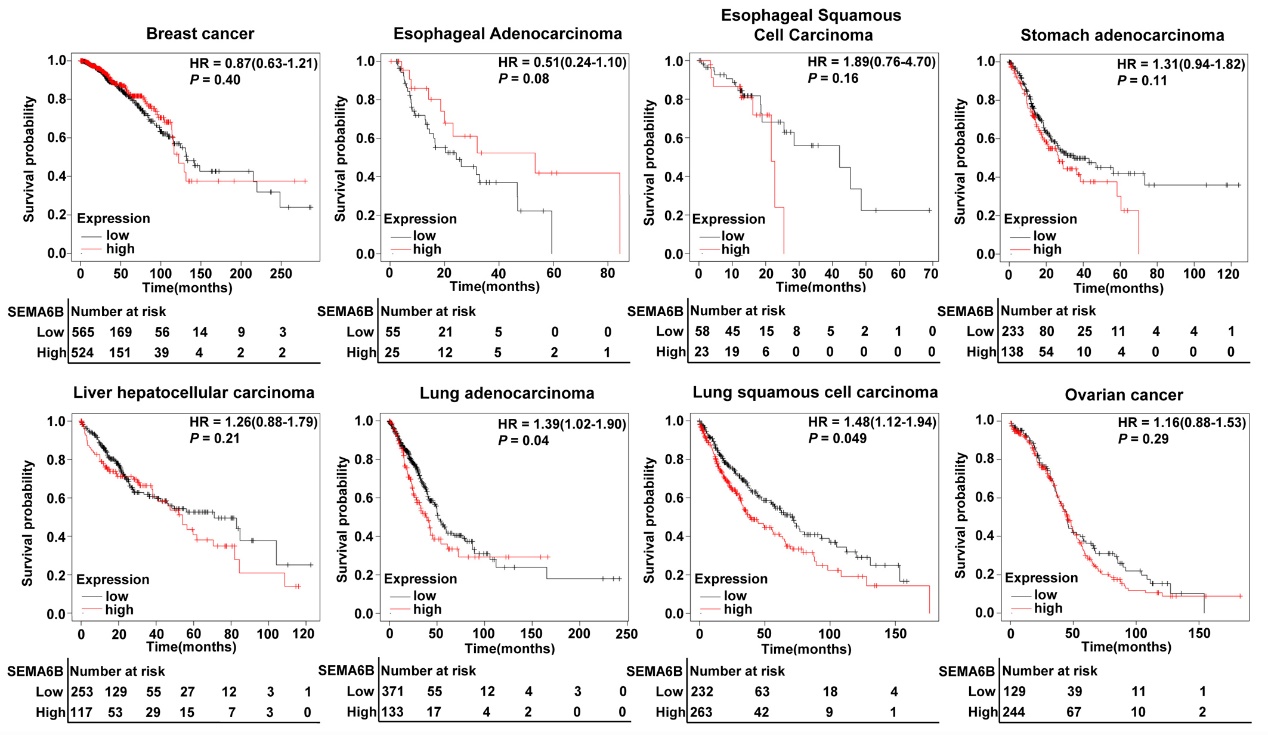


**Supplementary Figure S3** Kaplan-Meier survival curves of low and high SEMA6B expression groups divided by the best cut-off values in different types of human cancers from the TCGA database. The black line indicates the low SEMA6B expression group, and the red line indicates the high SEMA6B expression group.


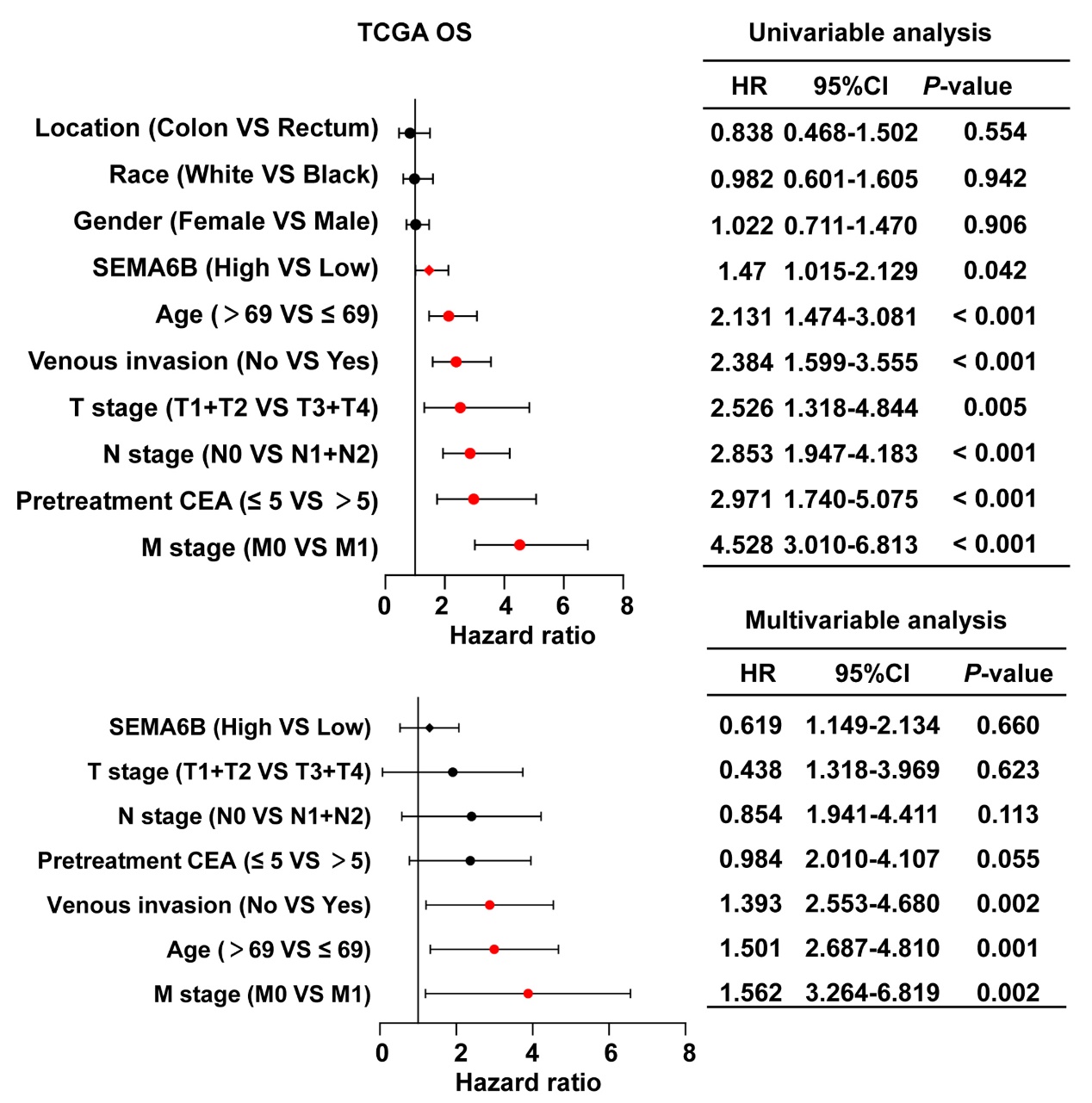


**Supplementary Figure S4** Univariate and multivariate analysis for overall survival (OS) calculated by Cox proportional hazard analysis in the TCGA CRC cohort. Forest plots visualizing hazard ratios (HRs) and corresponding 95% confidence intervals (CI) for each variable. Differences with P < 0.05 were considered significant.


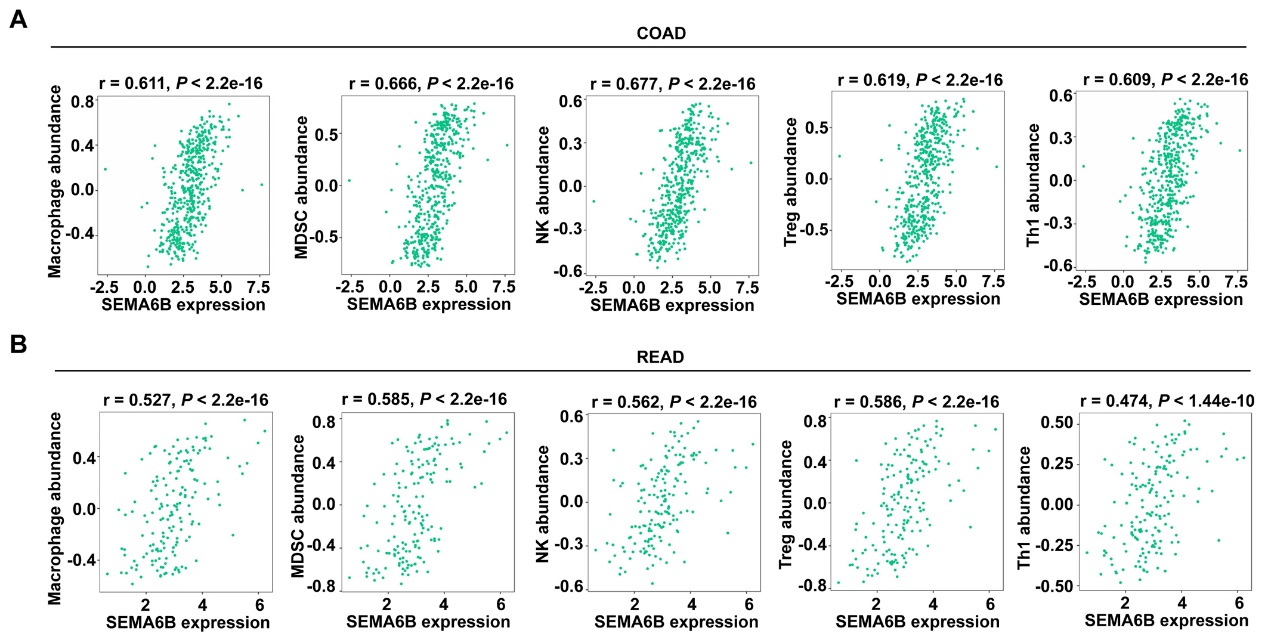


**Supplementary Figure S5** Correlations of SEMA6B expression with immune infiltrating levels in CRC patients from the TISIDB database. Scatter plots showing correlations of the top-five immune infiltrations with SEMA6B expression in COAD (n = 459) **(A)** and READ (n = 167) **(B)**. The Spearman correlation coefficient and corresponding *P* value are shown at the top of each plot.

**Supplementary Table S1** Stromal scores, immune scores, ESTIMATE scores, and tumor purities for each patient with CRC.

| ID | Stromal score | Immune score | ESTIMATE score | Tumor purity |
| --- | --- | --- | --- | --- |
| TCGA-3L-AA1B-01A | -1000.655 | -164.702 | -1165.357 | 0.907 |
| TCGA-4N-A93T-01A | -2182.903 | -750.845 | -2933.748 | 0.985 |
| TCGA-4T-AA8H-01A | -2400.820 | -935.633 | -3336.453 | 0.993 |
| TCGA-5M-AAT6-01A | -64.775 | 1104.736 | 1039.961 | 0.726 |
| TCGA-5M-AATE-01A | -1572.822 | -575.574 | -2148.396 | 0.958 |
| TCGA-A6-2671-01A | -271.765 | 159.836 | -111.929 | 0.832 |
| TCGA-A6-2672-01A | -326.554 | 1467.651 | 1141.096 | 0.716 |
| TCGA-A6-2674-01A | 716.640 | 1472.929 | 2189.569 | 0.601 |
| TCGA-A6-2675-01A | 86.481 | 623.079 | 709.560 | 0.759 |
| TCGA-A6-2676-01A | -572.264 | 1186.704 | 614.440 | 0.768 |
| TCGA-A6-2677-01A | -1845.325 | -792.969 | -2638.294 | 0.976 |
| TCGA-A6-2678-01A | -901.195 | 225.839 | -675.356 | 0.875 |
| TCGA-A6-2679-01A | -846.427 | 834.330 | -12.097 | 0.824 |
| TCGA-A6-2680-01A | -920.317 | 152.467 | -767.849 | 0.881 |
| TCGA-A6-2681-01A | -182.080 | 389.463 | 207.382 | 0.805 |
| TCGA-A6-2682-01A | -416.158 | 405.930 | -10.228 | 0.823 |
| TCGA-A6-2683-01A | -1480.528 | -329.591 | -1810.120 | 0.943 |
| TCGA-A6-2684-01A | -77.602 | 602.884 | 525.282 | 0.776 |
| TCGA-A6-2685-01A | 293.709 | 999.316 | 1293.024 | 0.700 |
| TCGA-A6-2686-01A | -201.614 | 1497.619 | 1296.005 | 0.700 |
| TCGA-A6-3807-01A | -611.417 | 492.786 | -118.631 | 0.832 |
| TCGA-A6-3808-01A | 149.884 | 891.074 | 1040.958 | 0.726 |
| TCGA-A6-3809-01A | -552.202 | 1038.614 | 486.411 | 0.780 |
| TCGA-A6-3810-01A | 60.437 | 588.427 | 648.864 | 0.765 |
| TCGA-A6-4105-01A | -368.760 | 736.318 | 367.558 | 0.791 |
| TCGA-A6-4107-01A | -526.738 | 411.558 | -115.180 | 0.832 |
| TCGA-A6-5656-01A | -1584.657 | -687.521 | -2272.178 | 0.963 |
| TCGA-A6-5657-01A | -354.142 | 315.007 | -39.135 | 0.826 |
| TCGA-A6-5659-01A | -922.623 | -550.011 | -1472.634 | 0.925 |
| TCGA-A6-5660-01A | -738.365 | -177.944 | -916.310 | 0.891 |
| TCGA-A6-5661-01A | -1297.971 | -68.848 | -1366.819 | 0.919 |
| TCGA-A6-5662-01A | -1083.119 | -596.065 | -1679.184 | 0.936 |
| TCGA-A6-5664-01A | 96.036 | 726.823 | 822.859 | 0.748 |
| TCGA-A6-5665-01A | -1734.391 | -78.072 | -1812.463 | 0.943 |
| TCGA-A6-5666-01A | -1819.028 | -663.766 | -2482.794 | 0.971 |
| TCGA-A6-5667-01A | -835.390 | -471.434 | -1306.824 | 0.916 |
| TCGA-A6-6137-01A | -1328.195 | 313.685 | -1014.510 | 0.898 |
| TCGA-A6-6138-01A | -185.187 | 1161.270 | 976.083 | 0.733 |
| TCGA-A6-6140-01A | -1715.259 | -246.712 | -1961.971 | 0.950 |
| TCGA-A6-6141-01A | -1024.251 | 452.476 | -571.776 | 0.867 |
| TCGA-A6-6648-01A | -1691.161 | -539.038 | -2230.200 | 0.962 |
| TCGA-A6-6649-01A | -568.064 | 335.170 | -232.894 | 0.841 |
| TCGA-A6-6650-01A | -1737.895 | -788.112 | -2526.007 | 0.973 |
| TCGA-A6-6651-01A | 849.574 | 1270.725 | 2120.300 | 0.609 |
| TCGA-A6-6652-01A | -1790.167 | -831.780 | -2621.947 | 0.976 |
| TCGA-A6-6653-01A | -943.796 | 123.410 | -820.386 | 0.885 |
| TCGA-A6-6654-01A | 738.748 | 1565.059 | 2303.808 | 0.587 |
| TCGA-A6-6780-01A | -974.331 | 1266.920 | 292.588 | 0.797 |
| TCGA-A6-6781-01A | 850.926 | 1221.801 | 2072.727 | 0.614 |
| TCGA-A6-6782-01A | -3.235 | 626.955 | 623.719 | 0.767 |
| TCGA-A6-A565-01A | -298.835 | 1398.901 | 1100.066 | 0.720 |
| TCGA-A6-A566-01A | 1231.211 | 1415.592 | 2646.803 | 0.546 |
| TCGA-A6-A567-01A | -1212.090 | -705.411 | -1917.500 | 0.948 |
| TCGA-A6-A56B-01A | -825.684 | -649.168 | -1474.851 | 0.925 |
| TCGA-A6-A5ZU-01A | -268.835 | 470.798 | 201.962 | 0.805 |
| TCGA-AA-3488-01A | -1533.759 | -182.188 | -1715.947 | 0.938 |
| TCGA-AA-3492-01A | -886.113 | 370.281 | -515.832 | 0.863 |
| TCGA-AA-3494-01A | -1377.564 | -132.493 | -1510.057 | 0.927 |
| TCGA-AA-3495-01A | -1243.913 | 257.737 | -986.176 | 0.896 |
| TCGA-AA-3502-01A | -1788.854 | 208.341 | -1580.513 | 0.931 |
| TCGA-AA-3506-01A | -804.240 | 515.907 | -288.333 | 0.846 |
| TCGA-AA-3509-01A | -1034.028 | 194.155 | -839.873 | 0.886 |
| TCGA-AA-3510-01A | -973.122 | 614.878 | -358.244 | 0.851 |
| TCGA-AA-3511-01A | -453.795 | -152.032 | -605.827 | 0.870 |
| TCGA-AA-3514-01A | -244.016 | 127.107 | -116.908 | 0.832 |
| TCGA-AA-3516-01A | -1061.688 | 501.325 | -560.363 | 0.866 |
| TCGA-AA-3517-01A | -1001.166 | 103.862 | -897.304 | 0.890 |
| TCGA-AA-3518-01A | -1192.847 | 617.577 | -575.269 | 0.868 |
| TCGA-AA-3519-01A | -1203.028 | 58.302 | -1144.727 | 0.906 |
| TCGA-AA-3520-01A | -423.724 | 277.818 | -145.905 | 0.835 |
| TCGA-AA-3522-01A | -1578.106 | 113.627 | -1464.479 | 0.925 |
| TCGA-AA-3524-01A | -1133.400 | -50.395 | -1183.795 | 0.908 |
| TCGA-AA-3525-01A | -1421.363 | 219.375 | -1201.988 | 0.910 |
| TCGA-AA-3526-01A | -845.795 | 482.374 | -363.421 | 0.852 |
| TCGA-AA-3530-01A | -1594.639 | 95.231 | -1499.408 | 0.927 |
| TCGA-AA-3531-01A | -1514.906 | -413.600 | -1928.506 | 0.949 |
| TCGA-AA-3532-01A | -411.112 | 971.404 | 560.292 | 0.773 |
| TCGA-AA-3534-01A | -1088.979 | -339.453 | -1428.432 | 0.923 |
| TCGA-AA-3538-01A | -749.689 | -135.900 | -885.589 | 0.889 |
| TCGA-AA-3542-01A | -1286.140 | -158.605 | -1444.745 | 0.924 |
| TCGA-AA-3543-01A | -724.768 | 1124.986 | 400.218 | 0.788 |
| TCGA-AA-3544-01A | -243.440 | 1197.610 | 954.170 | 0.735 |
| TCGA-AA-3548-01A | -910.550 | 373.343 | -537.207 | 0.865 |
| TCGA-AA-3549-01A | -1088.110 | 145.092 | -943.018 | 0.893 |
| TCGA-AA-3552-01A | -976.179 | 762.095 | -214.085 | 0.840 |
| TCGA-AA-3553-01A | -682.529 | 567.605 | -114.924 | 0.832 |
| TCGA-AA-3554-01A | -107.307 | 1191.437 | 1084.130 | 0.722 |
| TCGA-AA-3555-01A | -689.300 | -1.113 | -690.413 | 0.876 |
| TCGA-AA-3556-01A | -1199.507 | 124.694 | -1074.812 | 0.902 |
| TCGA-AA-3560-01A | -868.747 | 333.598 | -535.150 | 0.865 |
| TCGA-AA-3561-01A | -1347.662 | -123.277 | -1470.939 | 0.925 |
| TCGA-AA-3562-01A | -830.966 | 157.249 | -673.716 | 0.875 |
| TCGA-AA-3655-01A | -903.585 | -44.478 | -948.063 | 0.893 |
| TCGA-AA-3662-01A | -745.849 | 536.705 | -209.144 | 0.840 |
| TCGA-AA-3663-01A | -1313.379 | 67.647 | -1245.733 | 0.912 |
| TCGA-AA-3664-01A | -1672.110 | -179.076 | -1851.185 | 0.945 |
| TCGA-AA-3666-01A | -1150.232 | 329.352 | -820.880 | 0.885 |
| TCGA-AA-3667-01A | -1089.460 | 238.867 | -850.593 | 0.887 |
| TCGA-AA-3673-01A | -1086.840 | -36.039 | -1122.878 | 0.905 |
| TCGA-AA-3675-01A | -1263.572 | -193.259 | -1456.831 | 0.924 |
| TCGA-AA-3678-01A | -1295.203 | 375.605 | -919.597 | 0.892 |
| TCGA-AA-3679-01A | -1114.218 | 13.403 | -1100.815 | 0.903 |
| TCGA-AA-3680-01A | -1374.338 | 209.785 | -1164.553 | 0.907 |
| TCGA-AA-3681-01A | -1176.869 | 513.250 | -663.619 | 0.874 |
| TCGA-AA-3685-01A | -674.700 | 859.054 | 184.354 | 0.807 |
| TCGA-AA-3688-01A | -1250.388 | -36.629 | -1287.017 | 0.915 |
| TCGA-AA-3692-01A | -790.305 | 555.766 | -234.540 | 0.842 |
| TCGA-AA-3696-01A | -1241.152 | -631.085 | -1872.236 | 0.946 |
| TCGA-AA-3710-01A | -444.231 | 1640.117 | 1195.886 | 0.711 |
| TCGA-AA-3715-01A | 145.609 | 1383.850 | 1529.459 | 0.675 |
| TCGA-AA-3811-01A | -971.708 | 268.894 | -702.814 | 0.877 |
| TCGA-AA-3812-01A | -206.839 | 563.353 | 356.514 | 0.792 |
| TCGA-AA-3815-01A | -901.611 | 1221.055 | 319.444 | 0.795 |
| TCGA-AA-3818-01A | -1356.586 | -196.797 | -1553.383 | 0.930 |
| TCGA-AA-3819-01A | -1353.958 | -249.010 | -1602.968 | 0.932 |
| TCGA-AA-3821-01A | -696.730 | 171.669 | -525.060 | 0.864 |
| TCGA-AA-3831-01A | -1017.816 | 325.376 | -692.440 | 0.876 |
| TCGA-AA-3833-01A | -464.799 | 654.726 | 189.927 | 0.806 |
| TCGA-AA-3837-01A | -866.051 | -104.322 | -970.373 | 0.895 |
| TCGA-AA-3841-01A | -740.282 | 550.721 | -189.561 | 0.838 |
| TCGA-AA-3842-01A | -485.117 | 154.568 | -330.549 | 0.849 |
| TCGA-AA-3844-01A | -1398.315 | 133.968 | -1264.347 | 0.913 |
| TCGA-AA-3846-01A | -1148.764 | 351.693 | -797.071 | 0.883 |
| TCGA-AA-3848-01A | -1309.690 | -30.061 | -1339.751 | 0.918 |
| TCGA-AA-3851-01A | -964.472 | 487.727 | -476.745 | 0.860 |
| TCGA-AA-3854-01A | -1462.649 | -234.429 | -1697.078 | 0.937 |
| TCGA-AA-3855-01A | -1176.934 | 357.062 | -819.871 | 0.885 |
| TCGA-AA-3856-01A | -1053.432 | 832.688 | -220.744 | 0.841 |
| TCGA-AA-3858-01A | -798.148 | 41.794 | -756.355 | 0.880 |
| TCGA-AA-3860-01A | -417.479 | 583.703 | 166.224 | 0.808 |
| TCGA-AA-3861-01A | -1867.557 | 408.607 | -1458.949 | 0.925 |
| TCGA-AA-3862-01A | -1048.587 | 660.547 | -388.040 | 0.854 |
| TCGA-AA-3864-01A | -839.024 | -72.060 | -911.084 | 0.891 |
| TCGA-AA-3866-01A | -32.911 | 1253.106 | 1220.195 | 0.708 |
| TCGA-AA-3867-01A | -210.395 | 75.078 | -135.317 | 0.834 |
| TCGA-AA-3869-01A | -703.477 | 690.460 | -13.016 | 0.824 |
| TCGA-AA-3870-01A | -114.222 | 750.166 | 635.944 | 0.766 |
| TCGA-AA-3875-01A | -882.565 | 594.195 | -288.370 | 0.846 |
| TCGA-AA-3877-01A | -350.231 | 897.077 | 546.846 | 0.774 |
| TCGA-AA-3930-01A | -786.734 | 575.960 | -210.774 | 0.840 |
| TCGA-AA-3939-01A | -1006.580 | 362.542 | -644.038 | 0.873 |
| TCGA-AA-3941-01A | -1612.193 | -412.539 | -2024.732 | 0.953 |
| TCGA-AA-3947-01A | -385.942 | 508.348 | 122.406 | 0.812 |
| TCGA-AA-3949-01A | -51.273 | 1709.124 | 1657.850 | 0.661 |
| TCGA-AA-3950-01A | 83.257 | 1440.725 | 1523.982 | 0.676 |
| TCGA-AA-3952-01A | -518.776 | 46.330 | -472.446 | 0.860 |
| TCGA-AA-3955-01A | -1482.009 | -295.283 | -1777.291 | 0.941 |
| TCGA-AA-3956-01A | -829.308 | 151.722 | -677.586 | 0.875 |
| TCGA-AA-3966-01A | -22.139 | 1515.863 | 1493.724 | 0.679 |
| TCGA-AA-3968-01A | -531.874 | 240.678 | -291.196 | 0.846 |
| TCGA-AA-3970-01A | -1307.331 | 431.760 | -875.572 | 0.889 |
| TCGA-AA-3971-01A | -1051.796 | 676.791 | -375.004 | 0.853 |
| TCGA-AA-3972-01A | -1149.256 | -346.160 | -1495.415 | 0.927 |
| TCGA-AA-3973-01A | -1137.937 | -472.090 | -1610.027 | 0.933 |
| TCGA-AA-3975-01A | -875.167 | 181.041 | -694.126 | 0.876 |
| TCGA-AA-3976-01A | -997.255 | -126.611 | -1123.866 | 0.905 |
| TCGA-AA-3977-01A | -809.065 | 471.506 | -337.559 | 0.850 |
| TCGA-AA-3979-01A | -1683.616 | -540.283 | -2223.898 | 0.961 |
| TCGA-AA-3980-01A | -964.633 | 812.213 | -152.420 | 0.835 |
| TCGA-AA-3982-01A | -612.066 | 771.540 | 159.474 | 0.809 |
| TCGA-AA-3986-01A | -918.010 | 1181.906 | 263.897 | 0.800 |
| TCGA-AA-3989-01A | -512.234 | 600.988 | 88.754 | 0.815 |
| TCGA-AA-3994-01A | -918.811 | 51.935 | -866.876 | 0.888 |
| TCGA-AA-A004-01A | -780.783 | 572.305 | -208.478 | 0.840 |
| TCGA-AA-A00A-01A | -806.275 | 356.446 | -449.829 | 0.858 |
| TCGA-AA-A00D-01A | -471.854 | 1296.222 | 824.368 | 0.748 |
| TCGA-AA-A00E-01A | -927.696 | 717.344 | -210.353 | 0.840 |
| TCGA-AA-A00F-01A | -809.860 | -283.493 | -1093.353 | 0.903 |
| TCGA-AA-A00J-01A | -845.583 | -44.694 | -890.277 | 0.890 |
| TCGA-AA-A00K-01A | -1260.521 | -77.073 | -1337.594 | 0.918 |
| TCGA-AA-A00L-01A | -1677.663 | -602.653 | -2280.316 | 0.964 |
| TCGA-AA-A00N-01A | -99.786 | 291.977 | 192.191 | 0.806 |
| TCGA-AA-A00O-01A | -389.643 | -41.016 | -430.659 | 0.857 |
| TCGA-AA-A00Q-01A | -1194.275 | -172.557 | -1366.831 | 0.919 |
| TCGA-AA-A00R-01A | -663.535 | 1641.088 | 977.553 | 0.733 |
| TCGA-AA-A00U-01A | -1472.556 | -424.575 | -1897.131 | 0.947 |
| TCGA-AA-A00W-01A | -2252.812 | -401.276 | -2654.087 | 0.977 |
| TCGA-AA-A00Z-01A | -1456.699 | -306.057 | -1762.756 | 0.941 |
| TCGA-AA-A010-01A | -1351.627 | -55.335 | -1406.962 | 0.922 |
| TCGA-AA-A017-01A | -950.670 | -165.146 | -1115.816 | 0.904 |
| TCGA-AA-A01C-01A | -674.988 | -215.807 | -890.795 | 0.890 |
| TCGA-AA-A01D-01A | -299.909 | 309.793 | 9.884 | 0.822 |
| TCGA-AA-A01F-01A | -2215.571 | -588.067 | -2803.637 | 0.981 |
| TCGA-AA-A01G-01A | -1789.292 | -671.630 | -2460.922 | 0.970 |
| TCGA-AA-A01I-01A | -1733.057 | -229.802 | -1962.859 | 0.950 |
| TCGA-AA-A01K-01A | -556.367 | 156.838 | -399.530 | 0.854 |
| TCGA-AA-A01P-01A | -140.697 | 1444.286 | 1303.589 | 0.699 |
| TCGA-AA-A01Q-01A | -1519.510 | 255.896 | -1263.613 | 0.913 |
| TCGA-AA-A01R-01A | -1053.397 | 1163.635 | 110.237 | 0.813 |
| TCGA-AA-A01S-01A | -2136.982 | -923.676 | -3060.658 | 0.988 |
| TCGA-AA-A01V-01A | -2057.737 | 24.116 | -2033.622 | 0.953 |
| TCGA-AA-A01X-01A | -1338.452 | -361.948 | -1700.401 | 0.938 |
| TCGA-AA-A01Z-01A | -1746.418 | -838.564 | -2584.982 | 0.975 |
| TCGA-AA-A024-01A | -1794.912 | -416.606 | -2211.518 | 0.961 |
| TCGA-AA-A029-01A | -1870.889 | -589.014 | -2459.902 | 0.970 |
| TCGA-AA-A02E-01A | -1626.469 | -314.180 | -1940.649 | 0.949 |
| TCGA-AA-A02F-01A | -1449.905 | -1006.669 | -2456.574 | 0.970 |
| TCGA-AA-A02H-01A | -1273.190 | -446.000 | -1719.190 | 0.938 |
| TCGA-AA-A02J-01A | -2218.824 | -1079.002 | -3297.826 | 0.993 |
| TCGA-AA-A02K-01A | -1799.277 | -601.038 | -2400.314 | 0.968 |
| TCGA-AA-A02O-01A | -1491.332 | -128.262 | -1619.595 | 0.933 |
| TCGA-AA-A02R-01A | -553.890 | 1248.519 | 694.629 | 0.760 |
| TCGA-AA-A02W-01A | -1513.447 | -641.283 | -2154.730 | 0.959 |
| TCGA-AA-A02Y-01A | -2416.987 | -51.066 | -2468.053 | 0.971 |
| TCGA-AA-A03J-01A | -925.392 | 488.019 | -437.372 | 0.857 |
| TCGA-AD-5900-01A | -547.462 | 599.898 | 52.436 | 0.818 |
| TCGA-AD-6548-01A | -497.519 | 639.314 | 141.795 | 0.810 |
| TCGA-AD-6888-01A | -2454.425 | -687.526 | -3141.951 | 0.990 |
| TCGA-AD-6889-01A | -1501.403 | -356.296 | -1857.699 | 0.945 |
| TCGA-AD-6890-01A | -1059.230 | -190.881 | -1250.112 | 0.912 |
| TCGA-AD-6895-01A | -782.974 | 426.249 | -356.725 | 0.851 |
| TCGA-AD-6899-01A | 53.044 | 805.564 | 858.607 | 0.744 |
| TCGA-AD-6901-01A | 72.072 | 588.716 | 660.788 | 0.764 |
| TCGA-AD-6963-01A | -1434.696 | 339.823 | -1094.873 | 0.903 |
| TCGA-AD-6964-01A | 415.786 | 1597.361 | 2013.148 | 0.621 |
| TCGA-AD-6965-01A | -1293.462 | -546.580 | -1840.042 | 0.944 |
| TCGA-AD-A5EK-01A | -1391.768 | -755.165 | -2146.933 | 0.958 |
| TCGA-AF-2687-01A | 698.843 | 961.019 | 1659.862 | 0.661 |
| TCGA-AF-2690-01A | 1130.940 | 1751.309 | 2882.249 | 0.516 |
| TCGA-AF-2691-01A | -723.906 | 544.763 | -179.143 | 0.837 |
| TCGA-AF-2692-01A | -1064.620 | -110.343 | -1174.964 | 0.908 |
| TCGA-AF-2693-01A | -898.539 | 237.321 | -661.218 | 0.874 |
| TCGA-AF-3400-01A | 861.539 | 1891.118 | 2752.657 | 0.533 |
| TCGA-AF-3911-01A | -711.183 | -86.416 | -797.599 | 0.883 |
| TCGA-AF-3913-01A | -632.981 | -330.692 | -963.673 | 0.894 |
| TCGA-AF-4110-01A | -263.985 | 846.594 | 582.609 | 0.771 |
| TCGA-AF-5654-01A | -1682.580 | -775.756 | -2458.336 | 0.970 |
| TCGA-AF-6136-01A | -1318.206 | -315.151 | -1633.357 | 0.934 |
| TCGA-AF-6655-01A | -232.894 | -176.040 | -408.934 | 0.855 |
| TCGA-AF-6672-01A | -1569.112 | -376.347 | -1945.460 | 0.949 |
| TCGA-AF-A56K-01A | -224.716 | 217.112 | -7.605 | 0.823 |
| TCGA-AF-A56L-01A | -996.885 | -468.626 | -1465.511 | 0.925 |
| TCGA-AF-A56N-01A | -783.542 | -350.679 | -1134.221 | 0.905 |
| TCGA-AG-3574-01A | -1192.897 | -151.430 | -1344.327 | 0.918 |
| TCGA-AG-3575-01A | -184.440 | 613.559 | 429.119 | 0.785 |
| TCGA-AG-3578-01A | -1129.182 | 100.451 | -1028.731 | 0.899 |
| TCGA-AG-3580-01A | -1417.079 | 301.945 | -1115.134 | 0.904 |
| TCGA-AG-3581-01A | -953.768 | 210.831 | -742.937 | 0.880 |
| TCGA-AG-3582-01A | -793.090 | 132.363 | -660.727 | 0.874 |
| TCGA-AG-3583-01A | -1514.411 | 212.675 | -1301.736 | 0.916 |
| TCGA-AG-3584-01A | -709.234 | 177.876 | -531.358 | 0.864 |
| TCGA-AG-3586-01A | -1289.152 | 12.452 | -1276.701 | 0.914 |
| TCGA-AG-3587-01A | -996.387 | -29.013 | -1025.400 | 0.898 |
| TCGA-AG-3591-01A | -1471.684 | -35.395 | -1507.078 | 0.927 |
| TCGA-AG-3592-01A | -1000.117 | -52.728 | -1052.845 | 0.900 |
| TCGA-AG-3593-01A | -1121.912 | 489.725 | -632.187 | 0.872 |
| TCGA-AG-3594-01A | -1032.976 | 1162.883 | 129.907 | 0.812 |
| TCGA-AG-3598-01A | -1219.826 | 402.130 | -817.695 | 0.885 |
| TCGA-AG-3599-01A | -1392.497 | 250.809 | -1141.688 | 0.906 |
| TCGA-AG-3600-01A | -994.362 | 258.629 | -735.733 | 0.879 |
| TCGA-AG-3605-01A | -1596.174 | 146.198 | -1449.977 | 0.924 |
| TCGA-AG-3608-01A | -1024.071 | 584.785 | -439.287 | 0.857 |
| TCGA-AG-3609-01A | -283.504 | 402.812 | 119.308 | 0.812 |
| TCGA-AG-3611-01A | -1577.178 | 241.655 | -1335.523 | 0.918 |
| TCGA-AG-3612-01A | -518.745 | 224.797 | -293.948 | 0.846 |
| TCGA-AG-3726-01A | -812.585 | 112.751 | -699.834 | 0.876 |
| TCGA-AG-3727-01A | -667.868 | 23.779 | -644.089 | 0.873 |
| TCGA-AG-3728-01A | -114.638 | 1036.427 | 921.789 | 0.738 |
| TCGA-AG-3731-01A | 353.323 | 1009.505 | 1362.829 | 0.693 |
| TCGA-AG-3732-01A | -1240.042 | 656.428 | -583.614 | 0.868 |
| TCGA-AG-3742-01A | -827.757 | -439.216 | -1266.973 | 0.913 |
| TCGA-AG-3878-01A | 32.881 | 973.155 | 1006.036 | 0.730 |
| TCGA-AG-3881-01A | 204.403 | 967.582 | 1171.985 | 0.713 |
| TCGA-AG-3882-01A | -531.876 | 1273.559 | 741.682 | 0.756 |
| TCGA-AG-3883-01A | 152.755 | 797.971 | 950.726 | 0.735 |
| TCGA-AG-3885-01A | -1051.743 | 368.320 | -683.423 | 0.875 |
| TCGA-AG-3887-01A | -1101.753 | 165.825 | -935.929 | 0.893 |
| TCGA-AG-3890-01A | -875.133 | 575.774 | -299.358 | 0.847 |
| TCGA-AG-3892-01A | -1186.785 | 933.444 | -253.340 | 0.843 |
| TCGA-AG-3893-01A | -492.410 | 111.579 | -380.830 | 0.853 |
| TCGA-AG-3894-01A | -1186.039 | -143.652 | -1329.691 | 0.917 |
| TCGA-AG-3896-01A | -734.302 | 85.242 | -649.061 | 0.873 |
| TCGA-AG-3898-01A | -638.420 | 414.899 | -223.521 | 0.841 |
| TCGA-AG-3901-01A | 631.813 | 1131.092 | 1762.905 | 0.650 |
| TCGA-AG-3902-01A | -1145.160 | 955.024 | -190.136 | 0.838 |
| TCGA-AG-3909-01A | -1006.892 | 42.118 | -964.774 | 0.895 |
| TCGA-AG-3999-01A | -993.058 | -316.301 | -1309.359 | 0.916 |
| TCGA-AG-4001-01A | 7.342 | 344.008 | 351.350 | 0.792 |
| TCGA-AG-4005-01A | -699.481 | -243.219 | -942.700 | 0.893 |
| TCGA-AG-4007-01A | -537.373 | 519.093 | -18.280 | 0.824 |
| TCGA-AG-4008-01A | -227.285 | 134.086 | -93.199 | 0.830 |
| TCGA-AG-4021-01A | -342.345 | -226.496 | -568.841 | 0.867 |
| TCGA-AG-4022-01A | -284.931 | 501.075 | 216.144 | 0.804 |
| TCGA-AG-A002-01A | -1816.508 | -1026.954 | -2843.462 | 0.982 |
| TCGA-AG-A008-01A | -2068.187 | -484.566 | -2552.753 | 0.974 |
| TCGA-AG-A00C-01A | -2059.722 | -274.553 | -2334.275 | 0.966 |
| TCGA-AG-A00H-01A | -521.485 | -339.642 | -861.127 | 0.888 |
| TCGA-AG-A00Y-01A | -1628.194 | -4.827 | -1633.021 | 0.934 |
| TCGA-AG-A011-01A | -1128.844 | -206.872 | -1335.717 | 0.918 |
| TCGA-AG-A014-01A | -1517.953 | -596.070 | -2114.024 | 0.957 |
| TCGA-AG-A015-01A | -1923.649 | -635.491 | -2559.140 | 0.974 |
| TCGA-AG-A01J-01A | -1777.086 | -381.341 | -2158.427 | 0.959 |
| TCGA-AG-A01N-01A | -1521.898 | -752.139 | -2274.037 | 0.963 |
| TCGA-AG-A020-01A | -2083.068 | -776.772 | -2859.840 | 0.983 |
| TCGA-AG-A023-01A | -590.307 | 7.413 | -582.894 | 0.868 |
| TCGA-AG-A025-01A | -1179.729 | -339.303 | -1519.032 | 0.928 |
| TCGA-AG-A026-01A | -1260.458 | -827.053 | -2087.512 | 0.956 |
| TCGA-AG-A02G-01A | -1938.632 | -461.537 | -2400.168 | 0.968 |
| TCGA-AG-A02N-01A | -1837.780 | 53.778 | -1784.002 | 0.942 |
| TCGA-AG-A02X-01A | -1862.609 | -623.035 | -2485.644 | 0.971 |
| TCGA-AG-A032-01A | -1423.612 | -335.028 | -1758.641 | 0.940 |
| TCGA-AG-A036-01A | -1051.013 | -172.946 | -1223.959 | 0.911 |
| TCGA-AH-6547-01A | 45.566 | 1429.459 | 1475.025 | 0.681 |
| TCGA-AH-6549-01A | -635.584 | -240.531 | -876.114 | 0.889 |
| TCGA-AH-6643-01A | -1162.783 | -303.575 | -1466.357 | 0.925 |
| TCGA-AH-6644-01A | -178.451 | 97.826 | -80.625 | 0.829 |
| TCGA-AH-6897-01A | -1777.149 | -781.684 | -2558.833 | 0.974 |
| TCGA-AH-6903-01A | -1913.320 | -589.126 | -2502.446 | 0.972 |
| TCGA-AM-5820-01A | -999.067 | -455.422 | -1454.489 | 0.924 |
| TCGA-AM-5821-01A | -739.290 | 868.740 | 129.450 | 0.812 |
| TCGA-AU-3779-01A | -553.395 | 681.244 | 127.849 | 0.812 |
| TCGA-AU-6004-01A | -406.032 | 764.658 | 358.626 | 0.791 |
| TCGA-AY-4070-01A | -1070.513 | 10.219 | -1060.294 | 0.901 |
| TCGA-AY-4071-01A | -682.305 | 924.243 | 241.938 | 0.802 |
| TCGA-AY-5543-01A | -1612.925 | 122.795 | -1490.130 | 0.926 |
| TCGA-AY-6196-01A | 1395.071 | 2315.172 | 3710.244 | 0.409 |
| TCGA-AY-6197-01A | -1614.840 | -75.802 | -1690.641 | 0.937 |
| TCGA-AY-6386-01A | -1472.008 | 67.491 | -1404.517 | 0.922 |
| TCGA-AY-A54L-01A | -2524.453 | -1047.809 | -3572.262 | 0.997 |
| TCGA-AY-A69D-01A | -1526.786 | -574.895 | -2101.681 | 0.956 |
| TCGA-AY-A71X-01A | -2570.306 | -1053.872 | -3624.178 | 0.997 |
| TCGA-AY-A8YK-01A | -1579.336 | -481.590 | -2060.926 | 0.955 |
| TCGA-AZ-4308-01A | -314.930 | 244.916 | -70.014 | 0.828 |
| TCGA-AZ-4313-01A | -1438.199 | -415.223 | -1853.422 | 0.945 |
| TCGA-AZ-4315-01A | -915.268 | 280.102 | -635.166 | 0.872 |
| TCGA-AZ-4323-01A | -109.677 | 1707.369 | 1597.693 | 0.668 |
| TCGA-AZ-4614-01A | -2047.960 | -475.603 | -2523.564 | 0.973 |
| TCGA-AZ-4615-01A | -452.860 | 1013.254 | 560.394 | 0.773 |
| TCGA-AZ-4616-01A | -1080.313 | 168.508 | -911.805 | 0.891 |
| TCGA-AZ-4684-01A | -476.140 | 526.796 | 50.656 | 0.818 |
| TCGA-AZ-5403-01A | -408.113 | -75.832 | -483.945 | 0.861 |
| TCGA-AZ-5407-01A | -1810.816 | 92.501 | -1718.315 | 0.938 |
| TCGA-AZ-6598-01A | -1180.234 | 483.018 | -697.216 | 0.876 |
| TCGA-AZ-6599-01A | -2332.973 | -800.940 | -3133.913 | 0.990 |
| TCGA-AZ-6600-01A | -223.337 | 383.418 | 160.081 | 0.809 |
| TCGA-AZ-6601-01A | -337.131 | 1027.447 | 690.315 | 0.761 |
| TCGA-AZ-6603-01A | -426.361 | 284.891 | -141.470 | 0.834 |
| TCGA-AZ-6605-01A | 424.293 | 872.344 | 1296.638 | 0.700 |
| TCGA-AZ-6606-01A | -1911.629 | -536.433 | -2448.062 | 0.970 |
| TCGA-AZ-6607-01A | 576.319 | 696.803 | 1273.123 | 0.703 |
| TCGA-AZ-6608-01A | -2447.673 | -844.593 | -3292.266 | 0.993 |
| TCGA-BM-6198-01A | -456.520 | 830.246 | 373.726 | 0.790 |
| TCGA-CA-5254-01A | -846.963 | -432.814 | -1279.777 | 0.914 |
| TCGA-CA-5255-01A | -2078.947 | -722.053 | -2801.000 | 0.981 |
| TCGA-CA-5256-01A | -1175.272 | 113.659 | -1061.613 | 0.901 |
| TCGA-CA-5796-01A | -1653.382 | 44.014 | -1609.368 | 0.933 |
| TCGA-CA-5797-01A | -708.757 | -108.252 | -817.009 | 0.885 |
| TCGA-CA-6716-01A | -972.072 | -818.397 | -1790.469 | 0.942 |
| TCGA-CA-6717-01A | 273.469 | 1024.893 | 1298.362 | 0.700 |
| TCGA-CA-6718-01A | -627.799 | 1144.748 | 516.949 | 0.777 |
| TCGA-CA-6719-01A | -218.997 | 271.965 | 52.968 | 0.818 |
| TCGA-CI-6619-01B | -978.442 | 154.735 | -823.706 | 0.885 |
| TCGA-CI-6620-01A | -892.449 | -372.091 | -1264.541 | 0.913 |
| TCGA-CI-6621-01A | -459.165 | 454.849 | -4.315 | 0.823 |
| TCGA-CI-6622-01A | -1577.987 | -605.822 | -2183.809 | 0.960 |
| TCGA-CI-6623-01B | -1130.085 | -90.471 | -1220.556 | 0.911 |
| TCGA-CI-6624-01C | -365.881 | 448.786 | 82.905 | 0.816 |
| TCGA-CK-4947-01B | -611.326 | 587.951 | -23.374 | 0.824 |
| TCGA-CK-4948-01B | -492.743 | 70.569 | -422.174 | 0.856 |
| TCGA-CK-4950-01A | -1013.928 | 594.143 | -419.785 | 0.856 |
| TCGA-CK-4951-01A | -151.048 | 960.985 | 809.938 | 0.749 |
| TCGA-CK-4952-01A | -1405.361 | -252.364 | -1657.725 | 0.935 |
| TCGA-CK-5912-01A | -1137.916 | -485.936 | -1623.852 | 0.934 |
| TCGA-CK-5913-01A | -779.910 | 385.275 | -394.635 | 0.854 |
| TCGA-CK-5914-01A | -1024.658 | -209.437 | -1234.095 | 0.912 |
| TCGA-CK-5916-01A | -530.384 | 1047.640 | 517.256 | 0.777 |
| TCGA-CK-6747-01A | -1056.534 | -74.890 | -1131.423 | 0.905 |
| TCGA-CK-6748-01A | 45.355 | 205.308 | 250.663 | 0.801 |
| TCGA-CK-6751-01A | -562.318 | -91.841 | -654.159 | 0.873 |
| TCGA-CL-4957-01A | -1248.728 | -616.563 | -1865.291 | 0.946 |
| TCGA-CL-5917-01A | -1188.186 | -770.722 | -1958.907 | 0.950 |
| TCGA-CM-4743-01A | -1256.054 | 315.946 | -940.108 | 0.893 |
| TCGA-CM-4744-01A | -1669.865 | 312.605 | -1357.260 | 0.919 |
| TCGA-CM-4746-01A | -1749.685 | -320.921 | -2070.607 | 0.955 |
| TCGA-CM-4747-01A | -883.015 | -118.403 | -1001.418 | 0.897 |
| TCGA-CM-4748-01A | -612.700 | 149.918 | -462.782 | 0.859 |
| TCGA-CM-4750-01A | -1142.323 | -87.689 | -1230.012 | 0.911 |
| TCGA-CM-4751-01A | -727.560 | 541.277 | -186.283 | 0.838 |
| TCGA-CM-4752-01A | -611.206 | 630.628 | 19.422 | 0.821 |
| TCGA-CM-5341-01A | 254.509 | 1149.699 | 1404.209 | 0.689 |
| TCGA-CM-5344-01A | -488.067 | -440.596 | -928.663 | 0.892 |
| TCGA-CM-5348-01A | 331.226 | 709.281 | 1040.507 | 0.726 |
| TCGA-CM-5349-01A | -457.034 | 370.779 | -86.255 | 0.830 |
| TCGA-CM-5860-01A | 86.217 | 284.202 | 370.419 | 0.790 |
| TCGA-CM-5861-01A | -1632.286 | -358.669 | -1990.955 | 0.951 |
| TCGA-CM-5862-01A | -943.170 | -837.439 | -1780.609 | 0.942 |
| TCGA-CM-5864-01A | -1459.177 | -158.485 | -1617.662 | 0.933 |
| TCGA-CM-5868-01A | -817.709 | -346.307 | -1164.016 | 0.907 |
| TCGA-CM-6161-01A | -820.905 | 299.594 | -521.311 | 0.864 |
| TCGA-CM-6162-01A | 1005.299 | 1357.388 | 2362.686 | 0.580 |
| TCGA-CM-6163-01A | -469.636 | 474.584 | 4.947 | 0.822 |
| TCGA-CM-6164-01A | -1037.736 | -36.801 | -1074.537 | 0.902 |
| TCGA-CM-6165-01A | -297.675 | 200.124 | -97.551 | 0.831 |
| TCGA-CM-6166-01A | -1201.555 | -1017.221 | -2218.776 | 0.961 |
| TCGA-CM-6167-01A | 769.296 | 697.114 | 1466.410 | 0.682 |
| TCGA-CM-6168-01A | 431.000 | 794.090 | 1225.090 | 0.708 |
| TCGA-CM-6169-01A | 564.639 | 1260.024 | 1824.663 | 0.643 |
| TCGA-CM-6170-01A | -594.511 | 131.086 | -463.425 | 0.859 |
| TCGA-CM-6171-01A | -1241.535 | -3.270 | -1244.805 | 0.912 |
| TCGA-CM-6172-01A | -883.742 | -276.902 | -1160.644 | 0.907 |
| TCGA-CM-6674-01A | -593.635 | 350.296 | -243.339 | 0.842 |
| TCGA-CM-6675-01A | -1403.575 | -567.545 | -1971.120 | 0.951 |
| TCGA-CM-6676-01A | -861.941 | -684.512 | -1546.453 | 0.929 |
| TCGA-CM-6677-01A | -534.031 | 109.360 | -424.671 | 0.856 |
| TCGA-CM-6678-01A | -1289.843 | -701.480 | -1991.323 | 0.952 |
| TCGA-CM-6679-01A | -152.737 | 264.567 | 111.830 | 0.813 |
| TCGA-CM-6680-01A | -381.716 | 477.992 | 96.277 | 0.814 |
| TCGA-D5-5537-01A | -859.364 | -482.321 | -1341.685 | 0.918 |
| TCGA-D5-5538-01A | -135.086 | 883.656 | 748.571 | 0.755 |
| TCGA-D5-5539-01A | -385.096 | 314.981 | -70.116 | 0.828 |
| TCGA-D5-5540-01A | -1342.279 | -346.301 | -1688.580 | 0.937 |
| TCGA-D5-5541-01A | -488.520 | 365.537 | -122.983 | 0.833 |
| TCGA-D5-6529-01A | 56.755 | 1088.274 | 1145.029 | 0.716 |
| TCGA-D5-6530-01A | -1004.372 | 762.943 | -241.428 | 0.842 |
| TCGA-D5-6531-01A | -561.766 | 383.019 | -178.748 | 0.837 |
| TCGA-D5-6532-01A | -1634.006 | -576.435 | -2210.441 | 0.961 |
| TCGA-D5-6533-01A | -1051.056 | -145.715 | -1196.771 | 0.909 |
| TCGA-D5-6534-01A | 1090.928 | 1869.121 | 2960.049 | 0.507 |
| TCGA-D5-6535-01A | -979.131 | 302.960 | -676.171 | 0.875 |
| TCGA-D5-6536-01A | -651.589 | 164.698 | -486.891 | 0.861 |
| TCGA-D5-6537-01A | -1876.284 | -417.409 | -2293.693 | 0.964 |
| TCGA-D5-6538-01A | -1893.939 | -1272.486 | -3166.425 | 0.990 |
| TCGA-D5-6539-01A | -1273.399 | 63.200 | -1210.199 | 0.910 |
| TCGA-D5-6540-01A | -1102.968 | 163.098 | -939.870 | 0.893 |
| TCGA-D5-6541-01A | 65.962 | 839.975 | 905.937 | 0.740 |
| TCGA-D5-6898-01A | -359.893 | 280.232 | -79.660 | 0.829 |
| TCGA-D5-6920-01A | -1143.499 | 361.460 | -782.039 | 0.882 |
| TCGA-D5-6922-01A | -211.072 | -54.892 | -265.964 | 0.844 |
| TCGA-D5-6923-01A | -170.789 | -170.749 | -341.538 | 0.850 |
| TCGA-D5-6924-01A | 62.057 | 647.297 | 709.354 | 0.759 |
| TCGA-D5-6926-01A | 33.219 | 285.495 | 318.714 | 0.795 |
| TCGA-D5-6927-01A | -327.555 | 843.731 | 516.176 | 0.777 |
| TCGA-D5-6928-01A | 666.420 | 2656.785 | 3323.205 | 0.460 |
| TCGA-D5-6929-01A | -364.825 | 241.588 | -123.237 | 0.833 |
| TCGA-D5-6930-01A | -328.551 | 691.075 | 362.525 | 0.791 |
| TCGA-D5-6932-01A | -623.796 | 56.813 | -566.983 | 0.867 |
| TCGA-D5-7000-01A | -711.106 | 143.352 | -567.754 | 0.867 |
| TCGA-DC-4745-01A | -990.543 | -137.717 | -1128.260 | 0.905 |
| TCGA-DC-4749-01A | -1447.255 | -538.179 | -1985.434 | 0.951 |
| TCGA-DC-5337-01A | -1235.130 | 213.014 | -1022.116 | 0.898 |
| TCGA-DC-5869-01A | -796.482 | -340.316 | -1136.798 | 0.906 |
| TCGA-DC-6154-01A | -1219.408 | -401.825 | -1621.233 | 0.933 |
| TCGA-DC-6155-01A | -1107.303 | 92.210 | -1015.093 | 0.898 |
| TCGA-DC-6156-01A | 588.076 | 1214.084 | 1802.160 | 0.645 |
| TCGA-DC-6157-01A | -1174.742 | -440.953 | -1615.696 | 0.933 |
| TCGA-DC-6158-01A | 328.785 | 640.529 | 969.314 | 0.734 |
| TCGA-DC-6160-01A | -1440.370 | 181.669 | -1258.701 | 0.913 |
| TCGA-DC-6681-01A | -408.919 | -170.612 | -579.531 | 0.868 |
| TCGA-DC-6682-01A | -1648.269 | -516.778 | -2165.047 | 0.959 |
| TCGA-DC-6683-01A | -877.646 | -316.791 | -1194.437 | 0.909 |
| TCGA-DM-A0X9-01A | -1543.615 | -216.317 | -1759.932 | 0.941 |
| TCGA-DM-A0XD-01A | -1188.738 | -472.559 | -1661.297 | 0.935 |
| TCGA-DM-A0XF-01A | -1099.126 | -228.213 | -1327.339 | 0.917 |
| TCGA-DM-A1D0-01A | -2242.649 | -1183.015 | -3425.664 | 0.995 |
| TCGA-DM-A1D4-01A | -2228.071 | -496.296 | -2724.368 | 0.979 |
| TCGA-DM-A1D6-01A | -2110.485 | -1261.373 | -3371.858 | 0.994 |
| TCGA-DM-A1D7-01A | -1497.920 | -426.637 | -1924.558 | 0.948 |
| TCGA-DM-A1D8-01A | -1782.736 | -819.345 | -2602.081 | 0.975 |
| TCGA-DM-A1D9-01A | -1642.366 | -812.874 | -2455.240 | 0.970 |
| TCGA-DM-A1DA-01A | -1860.542 | -690.861 | -2551.403 | 0.974 |
| TCGA-DM-A1DB-01A | -2257.421 | -429.377 | -2686.797 | 0.978 |
| TCGA-DM-A1HA-01A | -2406.638 | -713.333 | -3119.971 | 0.989 |
| TCGA-DM-A1HB-01A | -1551.344 | -533.718 | -2085.062 | 0.956 |
| TCGA-DM-A280-01A | -1284.223 | -318.662 | -1602.885 | 0.932 |
| TCGA-DM-A282-01A | -1350.671 | -847.578 | -2198.249 | 0.960 |
| TCGA-DM-A285-01A | -895.891 | -947.165 | -1843.056 | 0.945 |
| TCGA-DM-A288-01A | -1942.289 | -895.486 | -2837.775 | 0.982 |
| TCGA-DM-A28A-01A | -1009.673 | -581.824 | -1591.496 | 0.932 |
| TCGA-DM-A28C-01A | -1955.382 | -967.823 | -2923.205 | 0.985 |
| TCGA-DM-A28E-01A | -2213.356 | -850.733 | -3064.089 | 0.988 |
| TCGA-DM-A28F-01A | -1752.865 | -593.262 | -2346.126 | 0.966 |
| TCGA-DM-A28G-01A | -1743.290 | -601.000 | -2344.290 | 0.966 |
| TCGA-DM-A28H-01A | -1768.257 | -978.473 | -2746.730 | 0.980 |
| TCGA-DM-A28K-01A | -1739.155 | -614.527 | -2353.682 | 0.967 |
| TCGA-DM-A28M-01A | -2292.297 | -732.764 | -3025.061 | 0.987 |
| TCGA-DT-5265-01A | -94.702 | 303.890 | 209.188 | 0.805 |
| TCGA-DY-A0XA-01A | -1399.199 | -600.124 | -1999.323 | 0.952 |
| TCGA-DY-A1DC-01A | -885.790 | -223.161 | -1108.951 | 0.904 |
| TCGA-DY-A1DD-01A | -1485.432 | -732.128 | -2217.561 | 0.961 |
| TCGA-DY-A1DE-01A | -1001.665 | -57.392 | -1059.057 | 0.901 |
| TCGA-DY-A1DF-01A | -1247.133 | -727.287 | -1974.420 | 0.951 |
| TCGA-DY-A1DG-01A | -2239.209 | -826.714 | -3065.923 | 0.988 |
| TCGA-DY-A1H8-01A | -2399.355 | -877.005 | -3276.360 | 0.992 |
| TCGA-EF-5830-01A | -1539.957 | -285.232 | -1825.189 | 0.944 |
| TCGA-EF-5831-01A | -608.966 | -196.869 | -805.834 | 0.884 |
| TCGA-EI-6506-01A | -1090.514 | 303.817 | -786.696 | 0.883 |
| TCGA-EI-6507-01A | -274.145 | 865.897 | 591.752 | 0.770 |
| TCGA-EI-6508-01A | -1263.828 | -597.545 | -1861.373 | 0.945 |
| TCGA-EI-6509-01A | -992.719 | -595.254 | -1587.973 | 0.932 |
| TCGA-EI-6510-01A | -2184.449 | -316.483 | -2500.932 | 0.972 |
| TCGA-EI-6511-01A | -241.067 | 1275.466 | 1034.399 | 0.727 |
| TCGA-EI-6512-01A | -1272.602 | -113.261 | -1385.863 | 0.920 |
| TCGA-EI-6513-01A | -1086.526 | -247.374 | -1333.900 | 0.917 |
| TCGA-EI-6514-01A | -660.264 | -229.106 | -889.370 | 0.890 |
| TCGA-EI-6881-01A | -1595.475 | -222.874 | -1818.349 | 0.943 |
| TCGA-EI-6882-01A | -1214.624 | -6.975 | -1221.599 | 0.911 |
| TCGA-EI-6883-01A | -1370.505 | -59.300 | -1429.805 | 0.923 |
| TCGA-EI-6884-01A | -525.220 | 453.300 | -71.919 | 0.828 |
| TCGA-EI-6885-01A | -253.732 | 298.763 | 45.031 | 0.819 |
| TCGA-EI-6917-01A | 50.158 | 1177.011 | 1227.169 | 0.707 |
| TCGA-EI-7002-01A | -641.066 | -148.782 | -789.848 | 0.883 |
| TCGA-EI-7004-01A | 1229.258 | 798.073 | 2027.331 | 0.620 |
| TCGA-F4-6459-01A | -5.587 | -82.162 | -87.748 | 0.830 |
| TCGA-F4-6460-01A | -383.675 | 42.321 | -341.355 | 0.850 |
| TCGA-F4-6461-01A | -434.242 | 236.173 | -198.069 | 0.839 |
| TCGA-F4-6463-01A | -354.817 | -173.663 | -528.480 | 0.864 |
| TCGA-F4-6569-01A | 806.839 | 727.257 | 1534.095 | 0.675 |
| TCGA-F4-6570-01A | 52.849 | 1296.707 | 1349.556 | 0.694 |
| TCGA-F4-6703-01A | 1291.059 | 2062.973 | 3354.033 | 0.456 |
| TCGA-F4-6704-01A | 617.587 | 429.763 | 1047.350 | 0.726 |
| TCGA-F4-6805-01A | 209.541 | 658.042 | 867.584 | 0.744 |
| TCGA-F4-6806-01A | -946.283 | -57.377 | -1003.660 | 0.897 |
| TCGA-F4-6807-01A | 458.591 | 975.947 | 1434.538 | 0.685 |
| TCGA-F4-6808-01A | -1746.198 | -648.452 | -2394.650 | 0.968 |
| TCGA-F4-6809-01A | -233.145 | 531.916 | 298.772 | 0.797 |
| TCGA-F4-6854-01A | -655.854 | -51.349 | -707.203 | 0.877 |
| TCGA-F4-6855-01A | 382.521 | 42.804 | 425.326 | 0.785 |
| TCGA-F4-6856-01A | -1336.631 | -179.460 | -1516.091 | 0.928 |
| TCGA-F5-6465-01A | 301.620 | 883.764 | 1185.384 | 0.712 |
| TCGA-F5-6571-01A | -45.204 | 776.686 | 731.482 | 0.757 |
| TCGA-F5-6702-01A | 833.338 | 436.703 | 1270.041 | 0.703 |
| TCGA-F5-6811-01A | 102.928 | 202.885 | 305.813 | 0.796 |
| TCGA-F5-6812-01A | 50.419 | 537.550 | 587.968 | 0.770 |
| TCGA-F5-6813-01A | -579.969 | 229.914 | -350.055 | 0.851 |
| TCGA-F5-6814-01A | -933.488 | 183.586 | -749.902 | 0.880 |
| TCGA-F5-6861-01A | -1285.708 | -450.510 | -1736.218 | 0.939 |
| TCGA-F5-6863-01A | -1234.230 | -987.039 | -2221.269 | 0.961 |
| TCGA-F5-6864-01A | 87.217 | 175.835 | 263.051 | 0.800 |
| TCGA-G4-6293-01A | -1146.876 | 617.862 | -529.014 | 0.864 |
| TCGA-G4-6294-01A | -1417.125 | -274.118 | -1691.243 | 0.937 |
| TCGA-G4-6295-01A | -1260.984 | 532.370 | -728.614 | 0.879 |
| TCGA-G4-6297-01A | -36.568 | 368.152 | 331.584 | 0.794 |
| TCGA-G4-6299-01A | -475.208 | 649.814 | 174.606 | 0.808 |
| TCGA-G4-6303-01A | -575.050 | -236.907 | -811.957 | 0.884 |
| TCGA-G4-6304-01A | -2051.729 | -204.559 | -2256.288 | 0.963 |
| TCGA-G4-6306-01A | -2339.540 | -330.068 | -2669.608 | 0.977 |
| TCGA-G4-6307-01A | -1998.721 | -837.552 | -2836.272 | 0.982 |
| TCGA-G4-6309-01A | -1618.469 | -329.771 | -1948.241 | 0.950 |
| TCGA-G4-6310-01A | -920.618 | -978.548 | -1899.167 | 0.947 |
| TCGA-G4-6311-01A | -500.501 | 212.044 | -288.457 | 0.846 |
| TCGA-G4-6314-01A | -63.084 | -161.712 | -224.796 | 0.841 |
| TCGA-G4-6315-01A | -2084.461 | -893.257 | -2977.719 | 0.986 |
| TCGA-G4-6317-01A | -2203.948 | -1203.871 | -3407.819 | 0.995 |
| TCGA-G4-6320-01A | -1947.753 | -497.563 | -2445.316 | 0.970 |
| TCGA-G4-6321-01A | -1800.476 | 424.630 | -1375.846 | 0.920 |
| TCGA-G4-6322-01A | -1279.074 | -264.035 | -1543.109 | 0.929 |
| TCGA-G4-6323-01A | -2098.768 | 540.774 | -1557.994 | 0.930 |
| TCGA-G4-6586-01A | -1914.862 | 339.102 | -1575.760 | 0.931 |
| TCGA-G4-6588-01A | -1111.663 | 67.919 | -1043.744 | 0.900 |
| TCGA-G4-6625-01A | -343.600 | 1268.668 | 925.068 | 0.738 |
| TCGA-G4-6626-01A | -1893.792 | -735.426 | -2629.217 | 0.976 |
| TCGA-G4-6627-01A | -189.178 | 761.433 | 572.255 | 0.772 |
| TCGA-G4-6628-01A | -535.808 | 1371.643 | 835.835 | 0.747 |
| TCGA-G5-6233-01A | -1142.728 | -205.062 | -1347.790 | 0.918 |
| TCGA-G5-6235-01A | -2239.376 | -697.298 | -2936.675 | 0.985 |
| TCGA-G5-6572-01A | -329.934 | -578.807 | -908.741 | 0.891 |
| TCGA-G5-6641-01A | -2460.904 | -1037.100 | -3498.004 | 0.996 |
| TCGA-NH-A50T-01A | -1803.663 | -1032.576 | -2836.239 | 0.982 |
| TCGA-NH-A50U-01A | -1191.011 | -524.189 | -1715.201 | 0.938 |
| TCGA-NH-A50V-01A | -473.704 | 206.984 | -266.720 | 0.844 |
| TCGA-NH-A6GA-01A | -1376.093 | -407.546 | -1783.639 | 0.942 |
| TCGA-NH-A6GB-01A | -1377.056 | 116.308 | -1260.748 | 0.913 |
| TCGA-NH-A6GC-01A | -693.218 | -506.446 | -1199.664 | 0.909 |
| TCGA-NH-A8F7-01A | -1958.764 | -1075.225 | -3033.990 | 0.987 |
| TCGA-NH-A8F8-01A | -707.361 | -162.995 | -870.356 | 0.888 |
| TCGA-QG-A5YV-01A | -1770.319 | -472.873 | -2243.192 | 0.962 |
| TCGA-QG-A5YW-01A | -1131.711 | 246.022 | -885.689 | 0.889 |
| TCGA-QG-A5YX-01A | -2106.274 | -644.976 | -2751.251 | 0.980 |
| TCGA-QG-A5Z1-01A | -768.099 | -537.825 | -1305.924 | 0.916 |
| TCGA-QG-A5Z2-01A | -2086.436 | 409.738 | -1676.698 | 0.936 |
| TCGA-QL-A97D-01A | -1647.711 | 271.643 | -1376.067 | 0.920 |
| TCGA-RU-A8FL-01A | -2634.537 | -1082.407 | -3716.944 | 0.998 |
| TCGA-SS-A7HO-01A | -1872.881 | -1104.007 | -2976.887 | 0.986 |
| TCGA-T9-A92H-01A | -1899.303 | -681.134 | -2580.437 | 0.975 |
| TCGA-WS-AB45-01A | 1608.685 | 1916.595 | 3525.280 | 0.433 |
